# Vibrational characteristics of aluminum–phosphate compounds by an experimental and theoretical approach

**DOI:** 10.1038/s41598-022-22432-5

**Published:** 2022-10-19

**Authors:** Pawel Goj, Bartosz Handke, Pawel Stoch

**Affiliations:** Faculty of Materials Science and Ceramics, AGH-University of Science and Technology, Al. Mickiewicza 30, 30-059 Kraków, Poland

**Keywords:** Inorganic chemistry, Materials chemistry, Theoretical chemistry

## Abstract

Aluminum phosphates are materials with relatively wide potential applications in many industries. The vibrational features of selected compounds were established on Raman and infrared spectroscopy. The experimentally determined spectra are compared to those calculated by ab initio methods. This gives a unique possibility of a proper assignment of the experimental spectral features to specific modes of vibration. In the results, it was evidenced that the spectra are characterized by two specific intense bands in the mid- and high-frequency range due to the P–O–P and P–O bonds in [PO_4_] tetrahedron vibrations. The position of the high-frequency band is related to the number of bridging oxygen atoms connecting [PO_4_] tetrahedrons in the unit cell. Additionally, the differences in the spectra were evidenced as a result of different polymorphic forms of the selected compounds. Therefore, the results may be useful in determining the phase composition of polyphase materials or structural features of aluminum–phosphate glasses and glass–ceramic materials.

## Introduction

Aluminum phosphates are present in many applications, for example in chemically bonded phosphate ceramics (CBPC) with alumina, dental cement, refractory binders, composite materials, and glass–ceramics^1–10^. Pyrophosphates containing aluminum and monovalent cations, such as NaAlP_2_O_7_, can be used as solid electrolytes for batteries, piezoelectric and ionic conductors^11–13^. Furthermore, NaAlP_2_O_7_ with different doped rare earth ions has potential application in white light emitting diodes (WLEDS)^14,15^. In the group of aluminum phosphates are molecular sieves (AlPO) that can be used in catalysis, separation, and ion exchange^16–18^.

Raman and infrared spectroscopies (IR), in addition to X-ray diffraction (XRD), are one of the most important methods of structural characterization of different materials. The spectroscopies are especially important in the case of amorphous materials such as glasses, where, because of the lack of long-range order, application of XRD is strongly limited. In this method, the proper assignment of characteristic bands to specific vibrations is a crucial point. To solve the problem, calculation methods based on density functional theory (DFT) can be very helpful. The methods allow for the prediction of theoretical IR and Raman spectra with considerable precision^19–23^.

The aim of the work was to compare theoretical and experimental IR and Raman spectra of different aluminum-phosphate compounds. Additionally, the theoretical results were used to determine the proper assignment of the characteristic spectral features to the different vibration modes. Special attention was paid to the position of the bands related to bond vibrations in the [PO_4_] tetrahedrons. The structural elements are the main building blocks of the aluminum phosphate compounds. Moreover, it is interesting to observe their changes resulting from structural transformations e.g. from chain to ring structures that may be evidenced in the compounds.

**Figure 1:**
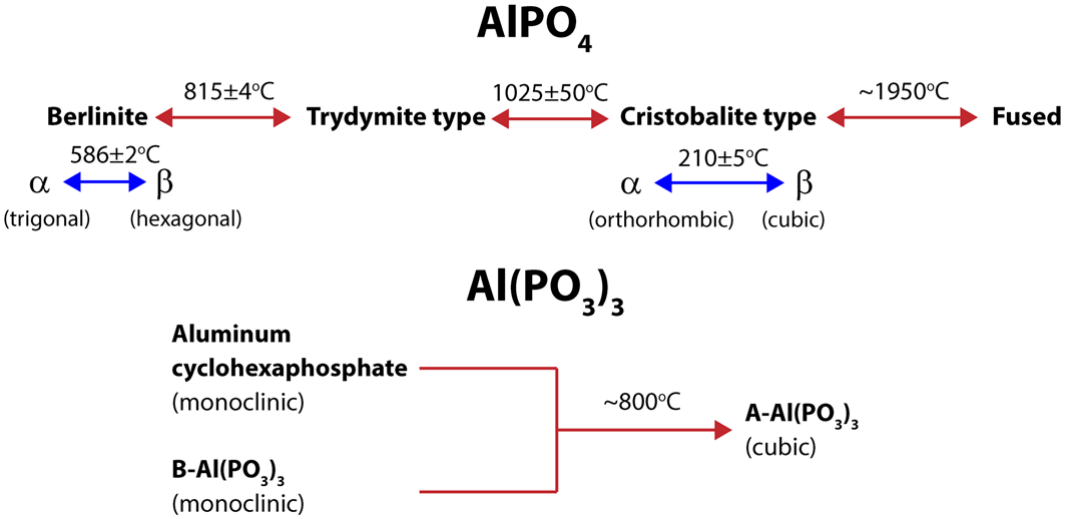
Thermal transformations of AlPO_4_ and Al(PO_3_)_3_. Crystal system in parentheses. Ref^4,28,30,33,38–41^.

In the work, the Q^i^ notation is applied as commonly used in phosphate glasses^19,24^. In this notation, ‘Q’ means phosphorus tetrahedron [PO_4_], and ‘i’ is a number of other phosphorus tetrahedra connected to ‘Q’. Aluminum phosphates were chosen so that all Q^i^ structural units were represented in the studies. Only the structural units Q^3^ are in pure P_2_O_5_, where the most stable polymorphic form is o’-P_2_O_5_^25^. Pure Q^2^ units are characteristic for Al(PO_3_)_3_, which has three polymorphs. A-Al(PO_3_)_3_ and aluminum cyklohexaphosphate, which have 4- and 6-membered [PO_4_] rings, respectively. Although B-Al(PO_3_)_3_ has a chain structure^4,26–28^. Aluminum cyklohexaphosphate and B-Al(PO_3_)_3_ are stable at temperatures lower than 800 °C but detailed studies have not been carried out. Above 800 °C, mainly A-Al(PO_3_)_3_ has been reported (Fig. 1)^4,28–30^. There is no pure aluminum–phosphate compound in which there are only Q^1^ units. Therefore, sodium-containing NaAlP_2_O_7_ was chosen. In the crystal structure, P_2_O_7_^4−^ dimers are present that are two joined Q^1^ structural units. The case of Q^0^ is represented by AlPO4, which is one of the most studied compounds of aluminum phosphates^4,7,31–37^. AlPO_4_ is a high refractory material with a melting point of about 1950 °C^38^ but a glaze on the surface due to probably the loss of P_2_O_5_ can be detected^32^. It undergoes several phase transformations, as shown in Fig. 1^39^, and the phases are isostructural to SiO_2_.

In the work low-temperature form of berlinite (α-berlinite) which is isostructural to α-quartz and low-temperature α-cristobalite type AlPO_4_ that has a close similar structure to α-cristobalite^31,33^ were studied.

Although the selected compounds are known, the number of literature data concerning their vibrational features is relatively limited. To the best of our knowledge, this is the first report in which all of the compounds are gathered together, and their experimental spectra are compared with the theoretical ones.

## Results

### o’-P_2_O_5_

**Figure 2:**
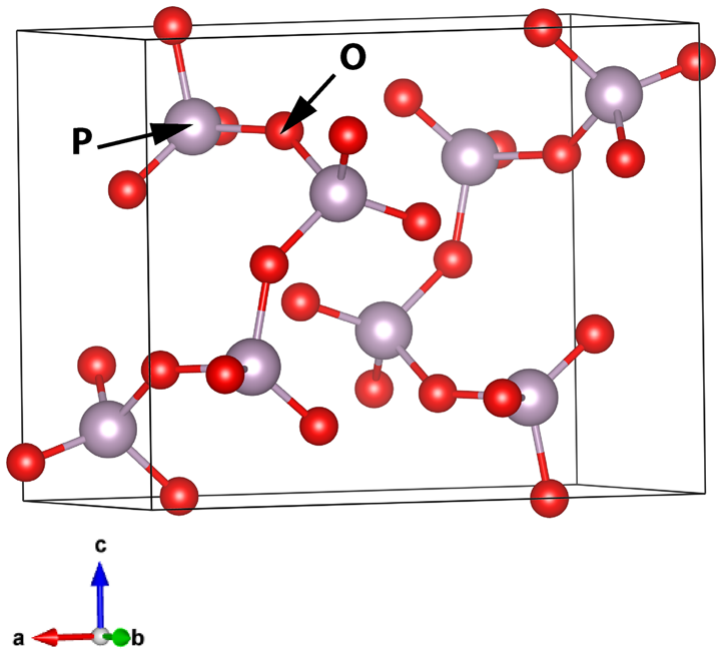
The unit cell of o’-P_2_O_5_ with optimized atom positions.

The DFT optimized unit cell of o’-P_2_O_5_ is shown in Fig. 2. In the unit cell, there exist only Q^3^ structural units. In the unit cell, 3 bridging oxygens are involved in the formation of P–O_B_–P bridges, and one is double-bonded to oxygen P=O. There are two inequivalent phosphorous sites with the mean P–O bond lengths 1.573 Å, 1.446 Å for P–O_B_ and P=O, respectively. The shorter length of the P=O bond leads to distortion of the [PO_4_] tetrahedron with the off-center shift of the central atom. The calculated Raman and IR intensities and their assignments are summarized in detail in Table S1.1 (supplementary materials). The calculated vibrations for o’-P_2_O_5_ were assigned to the vibrations of the idealized Q^3^ molecule (points group C_3v_) and idealized P-O_B_-P bridge (points group C_2v_). (Fig. S1.1 and S1.2 supplementary material).

**Figure 3:**
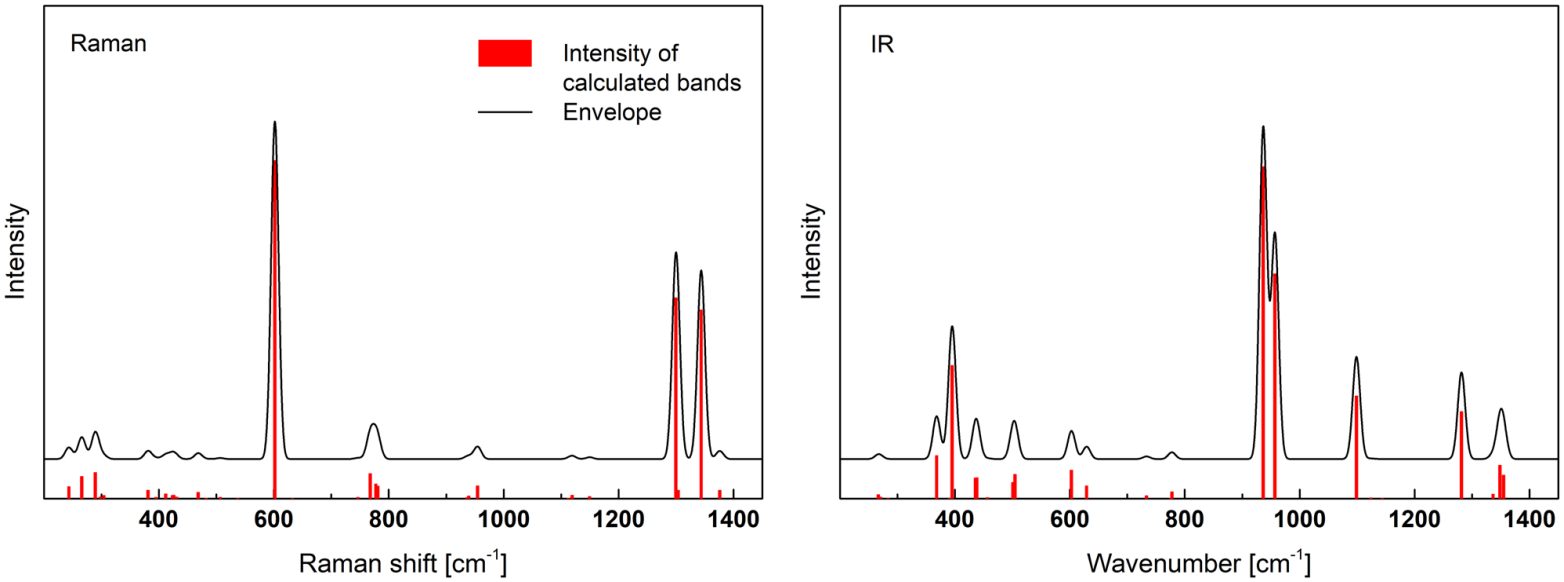
Calculated Raman and IR spectra of o’-P_2_O_5_.

Figure 3 shows the calculated Raman and IR spectra, and the simplified frequency ranges of the specific vibrations are summarized in Table 1. As can be seen in the Raman spectrum the most intense bands are at 604 cm^−1^ and 1300, 1344 cm^−1^. The lower frequency band is related to the symmetric (A_1_) and symmetric deformation (E) of 3(P-O_B_) in Q^3^. The higher value is due to the stretching of P=O in Q^3^ units. Other vibrations are considerably weaker. In the case of the IR spectrum, the strongest bands at 937 and 957 cm^−1^ are related to asymmetric stretching of P–O_B_–P and 3(P–O) in Q^3^. In this case, the bands due to P=O vibrations are also present, although their intensities are considerably lower.

**Table 1:** The calculated Raman and IR active modes of o’-P_2_O_5_ (more details in the supplementary material Table S1.1).

Frequency (> 200) [cm^−1^]	Intensity Raman (the most intense frequencies [cm^−1^])	Intensity IR (the most intense frequencies [cm^−1^])	Assignment to Q^i^ idealized vibrations and P–O_B_–P
< 300	Weak (244, 266 and 290 (s))	Very weak	Lattice vibrations and librations
368–483	Very weak	Medium (368, 395 (s), 435 and 438)	Asymmetric deformation (E) of 3(P-O_B_) in Q^3^
505–633	Very strong (602(s))	Weak (505, 603 (s))	Symmetric (A_1_) and asymmetric (E) deformation of 3(P-O_B_) in Q^3^ in different positions and Bending (A_1_) in P-O_B_-P
734–781	Weak (768, 778 and 781)	Very weak	Symmetric stretching (A_1_) in P-O_B_-P
936–939	Very weak	Very strong. (937 (s))	Asymmetric stretching (B_1_) in P-O_B_-P
955–957	Weak (955 (s))	Strong (957 (s))	Asymmetric stretching (E) of 3(P-O) in Q^3^
1099–1150	Very weak	Medium (1099 (s))	Symmetric (A_1_) and Asymmetric (E) stretching of 3(P-O_B_) in Q^3^ in different positions
1282–1376	Very strong (1300 (s) and 1344)	Medium (1282, 1349 (s) and 1355)	Symmetric stretching (A_1_) of P = O_NB_ in Q^3^

s—the strongest in range.

It should be noted that in this case, we present only theoretical spectra that were not scaled or shifted.

### B-Al(PO_3_)_3_

**Figure 4:**
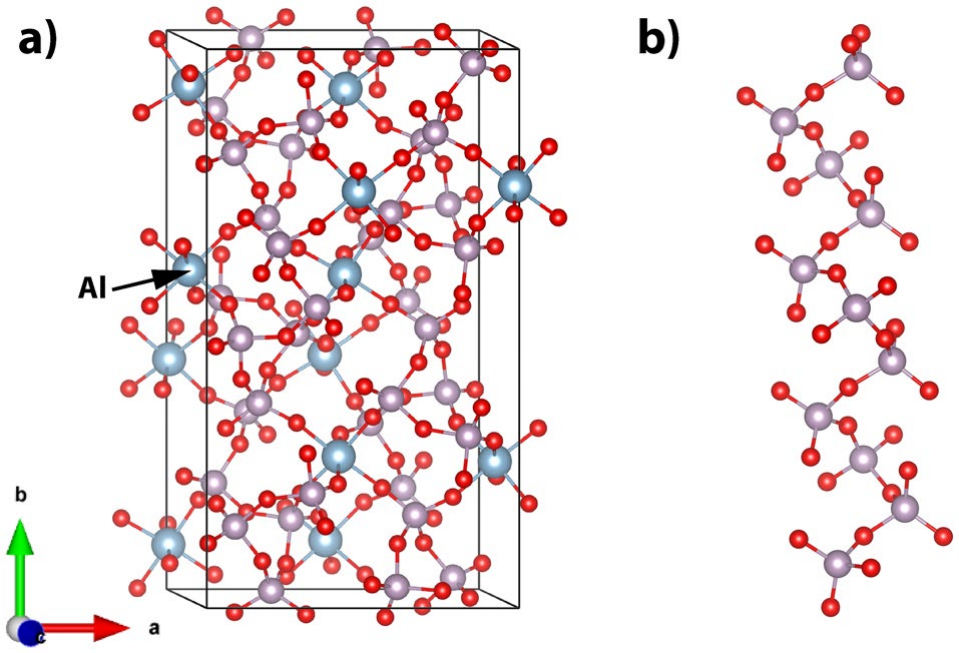
(**a**) Unit cell of B-Al(PO_3_)_3_ with the optimized atoms' positions and (**b**) chain of Q^2^ structural units.

The B-Al(PO_3_)_3_ is made up of infinitely twisted chains of structural units connected by [AlO_6_] octahedra. The unit cell is shown in Fig. 4. The length of the P-O_B_ bond changes in the range of 1.570–1.600 Å, whereas that of P-O_NB_ varies in the range of 1.480–1.488 Å. It should be pointed out that in the crystal structure there are no pure double-bonded oxygen atoms (P=O). All the non-bridging oxygens form P-O_NB_-Al bridges, and the excess phosphorus positive charge is redistributed over the two P-O_NB_ bonds.

**Figure 5:**
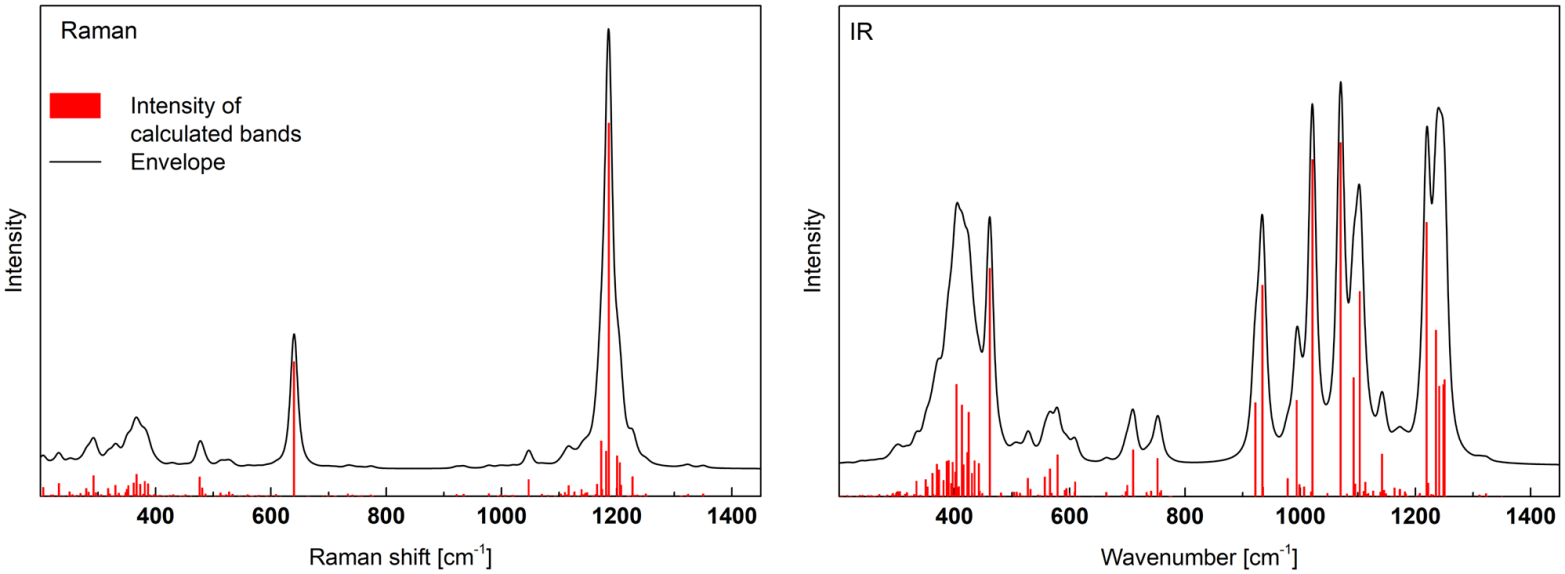
Calculated Raman and IR spectra of B-Al(PO_3_)_3_.

The calculated Raman and IR spectra are shown in Fig. 5. Detailed vibration assignments to idealized Q^2^ of the C_2v_ point group and their positions are summarized in Table S1.2 (supplementary material) and in simplification in Table 2.

**Table 2:** Calculated Raman and IR active modes of B-Al(PO_3_)_3_ (more details in the supplementary materials Table S1.2).

Frequency (> 200) [cm^−1^]	Intensity Raman (the most intense frequencies [cm^−1^])	Intensity IR (the most intense frequencies [cm^−1^])	Assignment to vibrations of Q^i^ idealized and P–O_B_–P
< 248	Very weak	Very weak	Lattice vibrations and librations
250–617	Weak (292, 367 (s) and 476)	Medium (389, 403 (s), 413, 422, 425, 435, 461 and 579)	Bending (A_1_) in P–O_B_–P and deformations (bending (A_1_) and asymmetric deformations (B_2_) of 2(P-O_NB_)) in Q^2^ (Q^2^-chains in [AlO_6_] environment)
640–777	Medium (640 (s))	Weak (710 (s) and 752)	Symmetric stretching (A_1_) in P-O_B_-P and bending (A_1_) of 2(P-O_NB_) in Q^2^ (Q^2^-chains in [AlO_6_] environment)
922–1021	Very weak	Strong (922, 934, 994 and 1021 (s))	Asymmetric stretching (B_1_) of 2(P-O_B_) in Q^2^ and asymmetric stretching (B_1_) in P-O_B_-P (Q^2^-chains in [AlO_6_] environment)
1047–1149	Very weak (1047 (s))	Strong (1070 (s), 1092, 1103 and 1142)	Symmetric stretching (A_1_) of 2(P-O_NB_) and symmetric stretching (A_1_) of 2(P-O_B_) in Q^2^ (Q^2^-chains in [AlO_6_] environment)
1159–1186	Very strong (1173, 1182 and 1186 (s))	Very weak	Symmetric stretching (A_1_) of 2(P–O_NB_) in Q^2^ (Q^2^-chains in [AlO_6_] environment)
1201–1350	Weak (1201 (s), 1206 and 1228)	Strong (1219 (s), 1236, 1241, 1249 and 1250)	Asymmetric stretching (B_1_) of 2(P–O_NB_) in Q^2^ (Q^2^-chains in [AlO_6_] environment)

s—the strongest in range.

The Raman spectrum of B-Al(PO_3_)_3_ is characterized by two intense bands. The strongest one is at 1186 cm^-1^ and weaker at 640 cm^−1^. The higher frequency band is related to the symmetric stretching vibrations (A_1_) of 2(P–O_NB_) in the Q^2^ structural units. The second lower frequency is due to symmetric stretching (A_1_) in P-O_B_-P and bending (A_1_) of 2(P-O_NB_) in Q^2^.

The IR spectrum of B-A(PO_3_)_3_ is more complex. In the range of 922–1350 cm^−1^, there are three groups of strong bands. The first group between 922 and 1021 cm^−1^ contains asymmetric stretching vibrations (B_1_) of 2(P-O_B_) in Q^2^ and asymmetric stretching (B_1_) in P-O_B_–P. The group between 1047 and 1149 cm^−1^ is related to the symmetric stretching (A_1_) of 2(P–O_NB_) and the symmetric stretching (A_1_) of 2(P–O_B_) in Q^2^. The last group between 1201 and 1350 cm^−1^ is due to the asymmetric stretching (B_1_) of 2(P–O_NB_) in Q^2^. The medium intensity in the IR spectrum has vibrations related to bending (A_1_) in P–O_B_–P and deformations in Q^2^ in the range of 250–617 cm^−1^. Other modes are much weaker.

### A-Al(PO_3_)_3_

**Figure 6:**
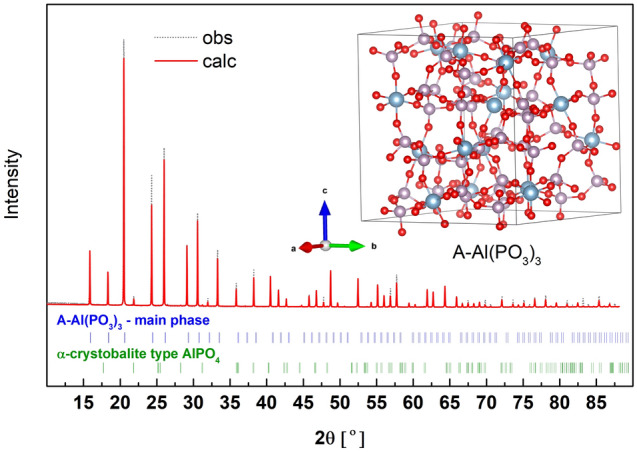
X-ray diffraction pattern (obs) and fit (calc) of A-Al(PO_3_)_3_. In the inset, the unit cell of A-Al(PO_3_)_3_ with the optimized atoms' positions.

The powder XRD diffraction pattern of the synthesized sample containing A-Al(PO_3_)_3_ is shown in Fig. 6. The Rietveld refinement of the data showed that in the sample two phases can be distinguished. The main crystalline phase is A-Al(PO_3_)_3_ in a quantity of approximately 99 wt% and the minority phase is an α-cristobalite type of AlPO_4_. The detailed composition of the material is given in Table S2.1 (supplementary data). The A-Al(PO_3_)_3_ crystallizes in a cubic I $\overline{4 }$ 3d space group and the fitted basic crystal structure parameter is a = 13.727(6) Å.

In contrast to B-Al(PO_3_)_3_ in the A-Al(PO_3_)_3_ phase the phosphate network forms 4-membered rings of Q^2^ structural units (4Q^2^ ring), and the rings are connected by polyhedrons [AlO_6_]. In this case, the length of the P-O_B_ bond is in the range of 1.583–1.595 Å, whereas for P-O_NB_ it is in the range of 1.471–1.479 Å. Similarly, as in B-Al(PO_3_)_3_ there are no pure double P=O bonds, and all the non-bridging oxygens take part in the formation of P-O_NB_-Al bridges.

**Figure 7:**
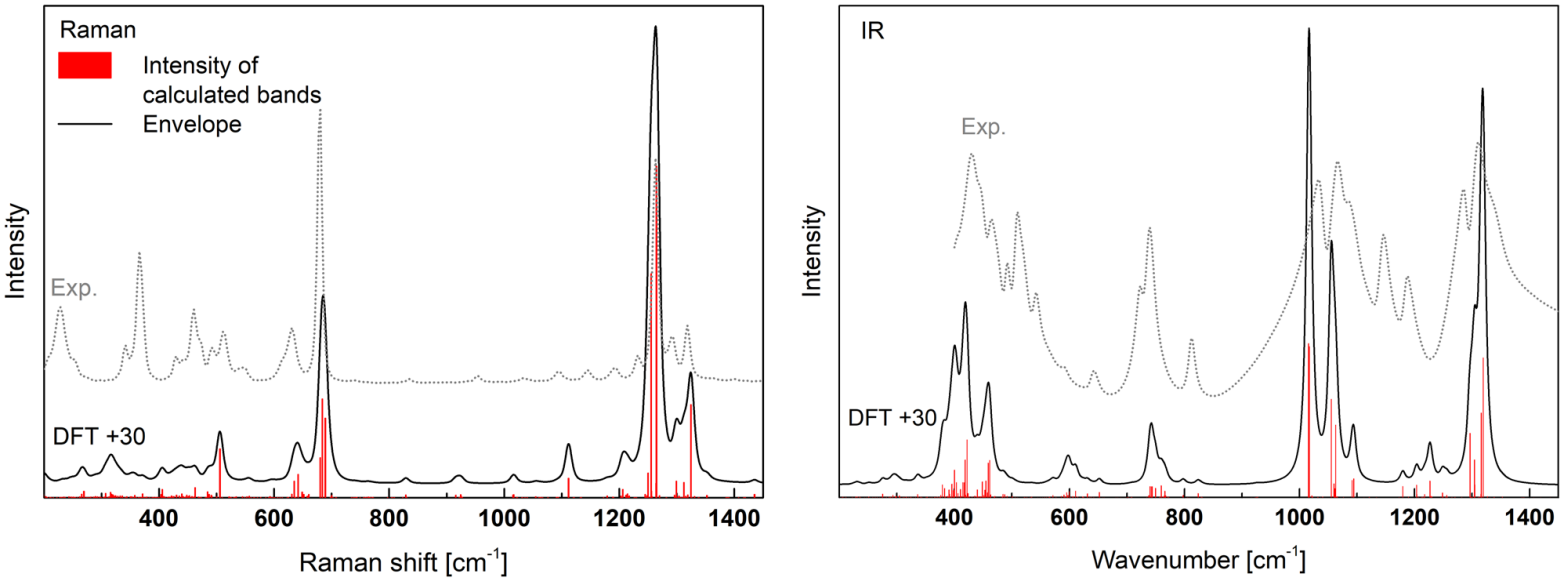
Calculated Raman and IR spectra of A-Al(PO_3_)_3_ shifted by + 30 cm^−1^ and experimental IR and Raman spectra (Exp.) of the synthesized material.

The calculated and experimental Raman and IR spectra are presented in Fig. 7. The corresponding vibrations are summarized in detail in Table S1.3 (supp.) and shortened in Table 3. The 4Q^2^ rings have S_4_ space group symmetry and some characteristic vibrations in A-Al(PO_3_)_3_ were assigned to this symmetry. It can be seen that there is a very good agreement between the experimental and theoretical results. In both the case of intensities and positions. However, the theoretical spectra were shifted by a constant value of about + 30 cm^−1^ for both Raman and IR results.

**Table 3:** The calculated Raman and IR active modes of A-Al(PO_3_)_3_ (more details in the supplementary materials Table S1.3).

Frequency (> 200) [cm^−1^]	Intensity Raman (the most intense frequencies [cm^−1^])	Intensity IR (the most intense frequencies [cm^−1^])	Assignment to Q^i^ idealized vibrations and P–O_B_–P
< 280	Very weak (236, 240 (s) and 277)	Very weak (245 (s))	Lattice vibrations and librations
286–651	Weak (286, 376, 433, 455, 476 (s), 605, 612, 619 and 651)	Medium (370, 389, 392 (s), 419, 429, 431 and 581)	Bending (A_1_) in P-O_B_-P and deformations (bending (A_1_) and asymmetric deformations (B_2_) of 2(P-O_NB_)) in Q^2^ (4Q^2^-rings in [AlO_6_] environment)
654–801	Strong (654 (s) and 659)	Medium (710, 714 and 730 (s))	Symmetric stretching (A_1_) in P-O_B_-P and bending (A_1_) of 2(P-O_NB_) in Q^2^ (4Q^2^-rings in [AlO_6_] environment)
886–1046	Very weak (799, 895 and 987 (s))	Strong (986 (s), 1025, 1030 and 1033)	Asymmetric stretching (B_1_) and symmetric stretching (A_1_) of 2(P-O_B_) in Q^2^ in different positions and asymmetric stretching (B_1_) in P-O_B_-P (4Q^2^-rings in [AlO_6_] environment)
1061–1149	Weak (1082 (s))	Medium (1061, 1064 (s) and 1149)	Symmetric stretching (A_1_) of 2(P-O_NB_) and symmetric stretching (A_1_) of 2(P-O_B_) in Q^2^ (4Q^2^-rings in [AlO_6_] environment)
1159–1240	Very strong (1176, 1220, 1226 (r) and 1235 (s))	Weak (1174, 1188, 1197 (s) and 1219)	Symmetric stretching (A_1_) of 2(P-O_NB_) in Q^2^ (4Q^2^-rings in [AlO_6_] environment) and asymmetric vibration about a fourfold inversion axis of 4Q^2^ ring
1252–1281	Weak (1270 (r, s))	Medium (1266 (s), 1274 and 1275)	Asymmetric stretching (B_1_) of 2(P-O_NB_) in Q^2^ (4Q^2^-rings in [AlO_6_] environment) and asymmetric vibration about the four-fold inversion axis of 4Q^2^ ring
1283–1405	Medium (1283 (r) and 1295 (r, s))	Strong (1286 and 1289 (s))	Asymmetric stretching (B_1_) of 2(P-O_NB_) in Q^2^ (4Q^2^-rings in [AlO_6_] environment) and symmetric vibration about a fourfold inversion axis of the 4Q^2^ ring

r—frequency assigned to the 4Q^2^ ring, s—the strongest in range.

**Figure 8:**
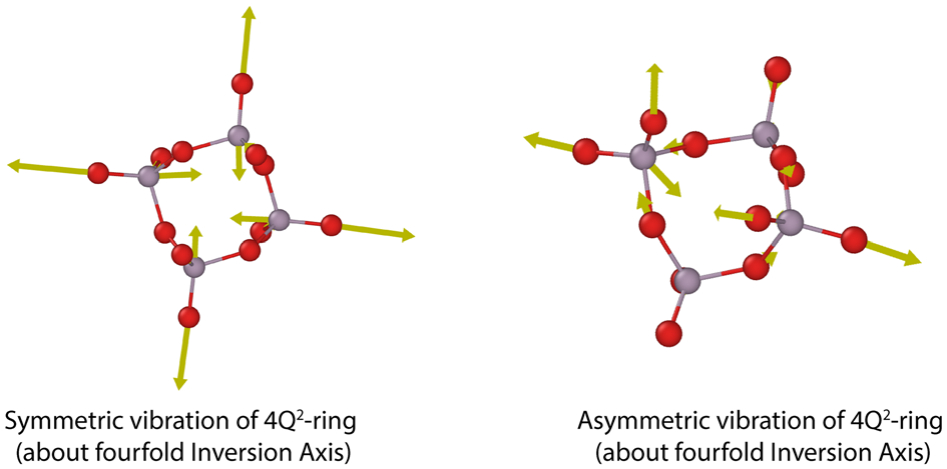
Examples of vibrations of 4Q^2^-rings in A-Al(PO_3_)_3_.

The Ramana spectrum of A-Al(PO_3_)_3_ has a very strong band at around 1235 cm^−1^ due to the symmetric stretching (A_1_) of 2(P-O_NB_) in Q^2^. The second band of lower intensity at 654 cm^−1^ is related to symmetric stretching (A_1_) in P–O_B_–P and bending (A_1_) of 2(P-O_NB_) in Q^2^. In the case of the studied phase, there exist characteristic ring vibrations as presented in Fig. 8. Two bands related to the vibrations at c.a. 1270 cm^−1^ and 1295 cm^−1^, which is due to asymmetrical and symmetrical vibrations about a fourfold inversion axis of the 4Q^2^ ring, respectively. It should be noted that the vibrations are characteristic for A-Al(PO_3_)_3_ and are not present in B-Al(PO_3_)_3_. Thus, it can be used to distinguish between the two phases.

The IR spectrum has two groups with strong bands at c.a. 886–1046 cm^−1^ and 1283–1405 cm^−1^. The first group is related to the stretching modes 2(P–O_B_) in Q^2^ and asymmetric stretching (B_1_) in P–O_B_–P. The band group 1283–1405 cm^-1^ is related to asymmetric stretching vibrations (B_1_) of 2(P-O_NB_) in Q^2^. The bands related to the bending modes (A1) in P–O_B_–P and deformations of 2(P-O_NB_)) in Q^2^ are in the range of 286–651 cm^−1^ and have a medium intensity. Also, visible in the IR spectra are bands related to symmetric stretching (A_1_) in P-O_B_-P and bending (A_1_) of 2(P-O_NB_) in Q^2^ in the range of 654–801 cm^−1^.

### Aluminum cyclohexaphosphate—Al(PO_3_)_3_

**Figure 9:**
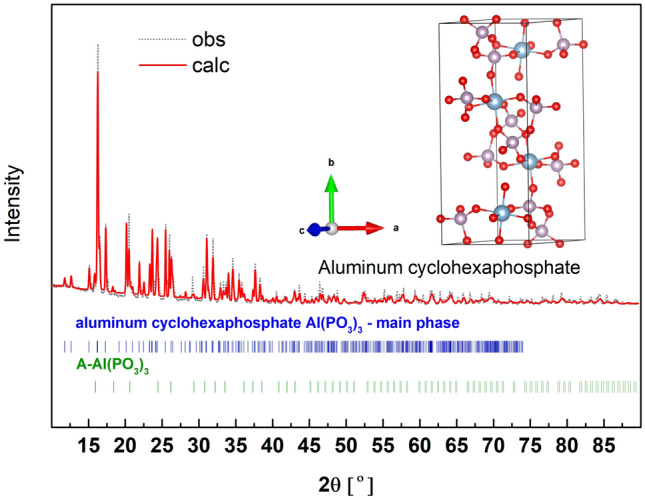
X-ray diffraction pattern (obs) and the fitted (calc) of aluminum cyclohexaphosphate -Al(PO_3_)_3_. In the inset, the unit cell of the compound with the optimized atom positions.

Another polymorphic form of Al(PO_3_)_3_ is aluminum cyclohexaphosphate. The powder X-ray diffraction pattern of the synthesized material is shown in Fig. 9. According to the Rietveld analysis, the assumed phase is the main (c.a. 85 wt%) and the rest is A-Al(PO_3_)_3_. The detailed phase composition of the material is summarized in Table S2.2 (supp.). The main phase crystallizes in a monoclinic P12_1_/c1 space group and the fitted crystal structure parameters are a=6.072(2) Å, b = 15.036(1) Å, c = 8.182(9) Å, *β *= 105.12°.

The crystal structure of the aluminum cyclohexaphosphate Al(PO_3_)_3_ is similar to A-Al(PO_3_)_3_ built of rings that, on the contrary, are composed of 6Q^2^ units connected by [AlO_6_] octahedra. In this case, the length of the P-O_B_ bond is in the range of 1.581–1.598 Å, whereas for P-O_NB_ it is in the range of 1.475–1.488 Å.

**Figure 10:**
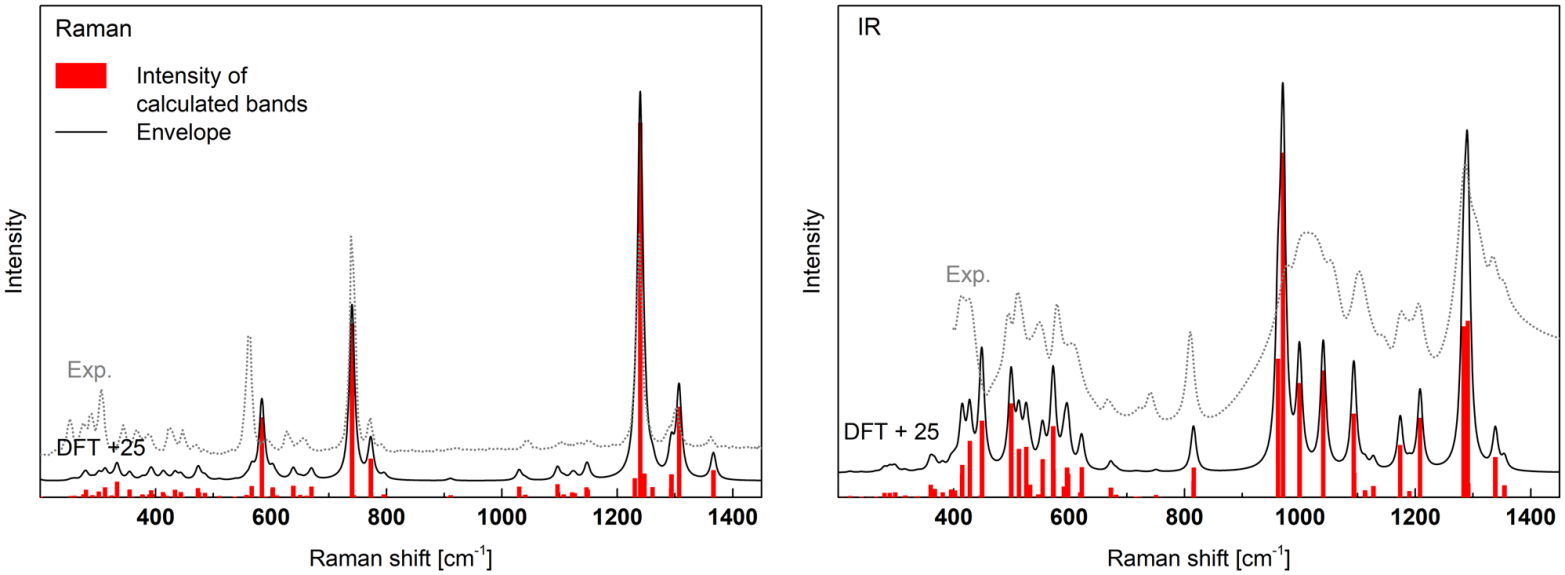
Calculated Raman and IR spectra of aluminum cyclohexaphosphate -Al(PO_3_)_3_ shifted by + 25 cm^−1^ and experimental IR and Raman spectra (Exp.) of the synthesized material.

The calculated and experimental Raman and IR spectra are presented in Fig. 10. The corresponding vibrations are summarized in detail in Table S1.4 (supp.) and shortened in Table 4. The 6Q^2^ rings have C_i_ space-group symmetry and some characteristic vibrations were assigned to this symmetry. Similarly to previously, there is good agreement between the experimental and theoretical results. The best convergence is obtained when the theoretical spectrum is shifted by the constant value of c.a. + 25 cm^-1^.

**Table 4:** The calculated Raman and IR active modes of aluminum cyclohexaphosphate -Al(PO3)3 (more details in the supplementary materials Table S1.4).

Frequency (> 200) [cm^−1^]	Intensity Raman (the most intense frequencies [cm^−1^])	Intensity IR (the most intense frequencies [cm^−1^])	Assignment to Q^i^ idealized vibrations and P–O_B_–P
< 260	Very weak	Very weak	Lattice vibrations and librations
265–555	Very weak (287, 308 (s), 448 and 541)	Medium (389, 402, 423, 474 (s), 488, 500, 529 and 547)	Bending (A_1_) in P-O_B_-P and deformations (bending (A_1_) and asymmetric deformations (B_2_) of 2(P-O_NB_)) in Q^2^ (6Q^2^-rings in [AlO_6_] environment)
559 (r)	Medium	Nonactive	Twisting (A_2_) of 2(P-O_NB_) and 2(P-O_B_) in Q^2^ (6Q^2^-rings in [AlO_6_] environment) and symmetric A_g_ vibration of 6Q^2^ molecule
565–656	Weak (578, 613 (s) and 645)	Medium	
(571 and 597 (s))	Bending (A_1_) in P-O_B_-P and deformations (bending (A_1_) and asymmetric deformations (B_2_) of 2(P-O_NB_)) in Q^2^ (6Q^2^-rings in [AlO_6_] environment)		
690–791	Strong (715 (s) and 748)	Weak (791(s))	Symmetric stretching (A1) in P-O_B_-P and bending (A_1_) of 2(P-O_NB_) in Q^2^ (6Q^2^-rings in [AlO_6_] environment)
883–1082	Weak (1005 and 1071 (s))	Strong (937, 946 (s), 974, 1015 and 1068)	Symmetric (A_1_) and asymmetric (B_1_) stretching of 2(P-O_B_) in different Q^2^ positions and asymmetric stretching (B_1_) in P-O_B_-P (6Q^2^-rings in [AlO_6_] environment)
1088–1215	VERY strong (1121 (r), 1205 (r, s) and 1215 (r))	Medium (1149 (r) and 1183 (r, s))	Symmetric stretching (A_1_) of 2(P-O_NB_) in the Q^2^ (6Q^2^-rings in [AlO_6_] environment) and Symmetric (Raman spectra) A_g_ and asymmetric (IR spectra) A_u_ vibration of 6Q^2^ molecule
1222–1341	Medium (1222 (r), 1236 (r), 1268 (r), 1282 (r) and 1341 (r, s))	Strong (1259, 1265 (s) and 1314)	Asymmetric stretching (B_1_) of 2(P-O_NB_) in Q^2^ (6Q^2^-rings in [AlO_6_] environment) and symmetric A_g_ vibration of 6Q^2^ molecule

r—frequency assigned to the 6Q^2^ ring, s—the strongest in the range.

**Figure 11:**
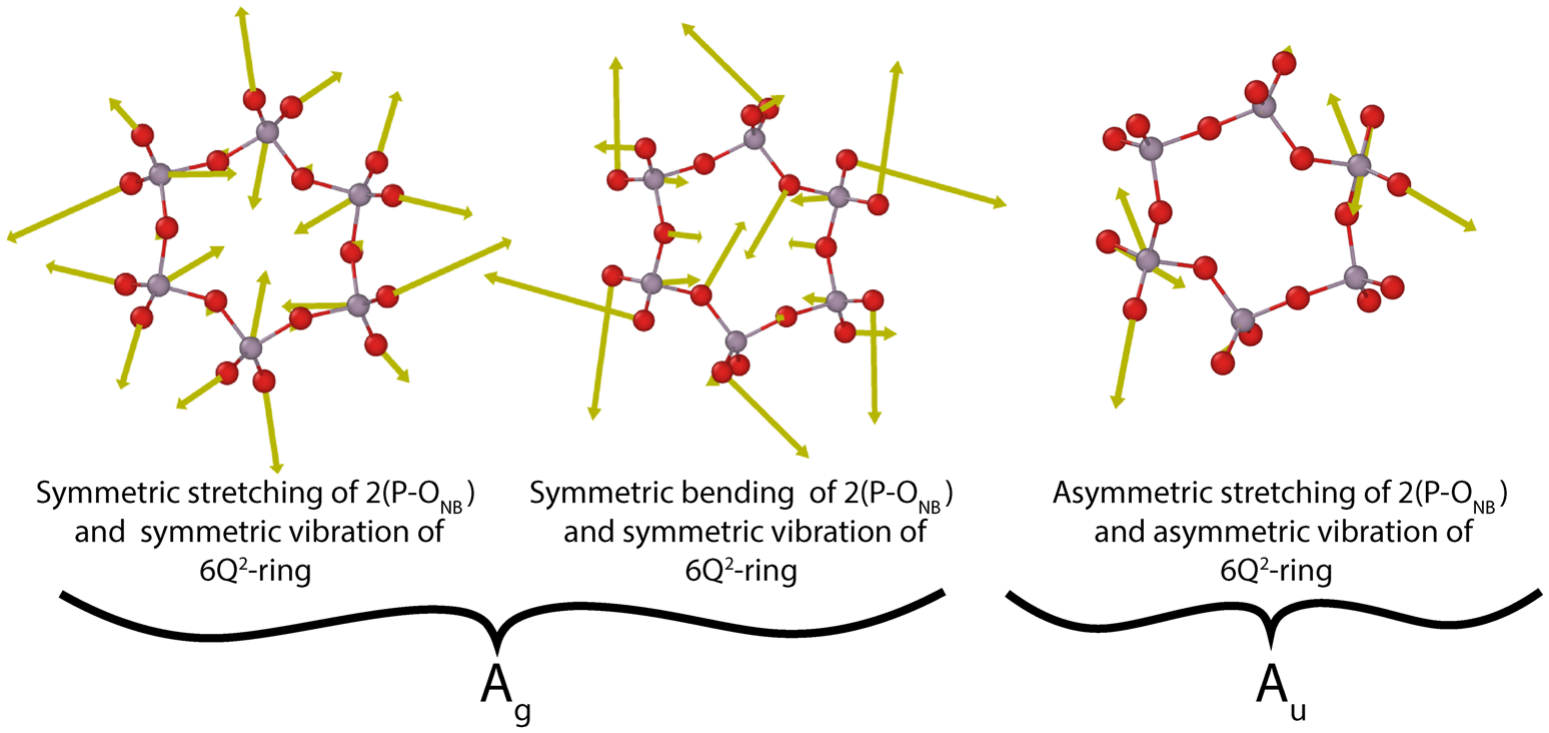
Examples of vibrations of 6Q^2^-ring in aluminum cyclohexaphosphate -Al(PO_3_)_3_.

The Raman spectrum of aluminum cyclohexaphosphate is similar to those of A-Al(PO_3_)_3_ and B-Al(PO_3_)_3_. The strongest band is related to the symmetric stretching modes (A_1_) of 2(P-O_NB_) in Q^2^. The position of the band is c.a. 1215 cm^−1^. The second strong band is at c.a. 715 cm^-1^ and is due to symmetric stretching vibrations (A_1_) in P-O_B_-P and bending modes (A_1_) of 2(P-O_NB_) in Q^2^. There are also characteristic 6Q^2^-ring modes active like symmetric vibrations A_g_ (Fig. 11) in the range of 1121–1341 cm^−1^ and 561 cm^−1^.

In the IR spectrum, the strongest vibrations are in the range of 883–1082 and 1222–1341 cm^−1^. The first group is related to the stretching of 2(P–O_B_) Q^2^ and the asymmetric stretching (B_1_) in P–O_B_–P. The second group is related to the asymmetric stretching (B_1_) of 2(P-O_NB_) in Q^2^. Good visible vibrations in the range 1088–1215 cm^-1^ are related to the asymmetric A_u_ vibration of the 6Q^2^-ring (Fig. 11).

### NaAlP_2_O_7_

**Figure 12:**
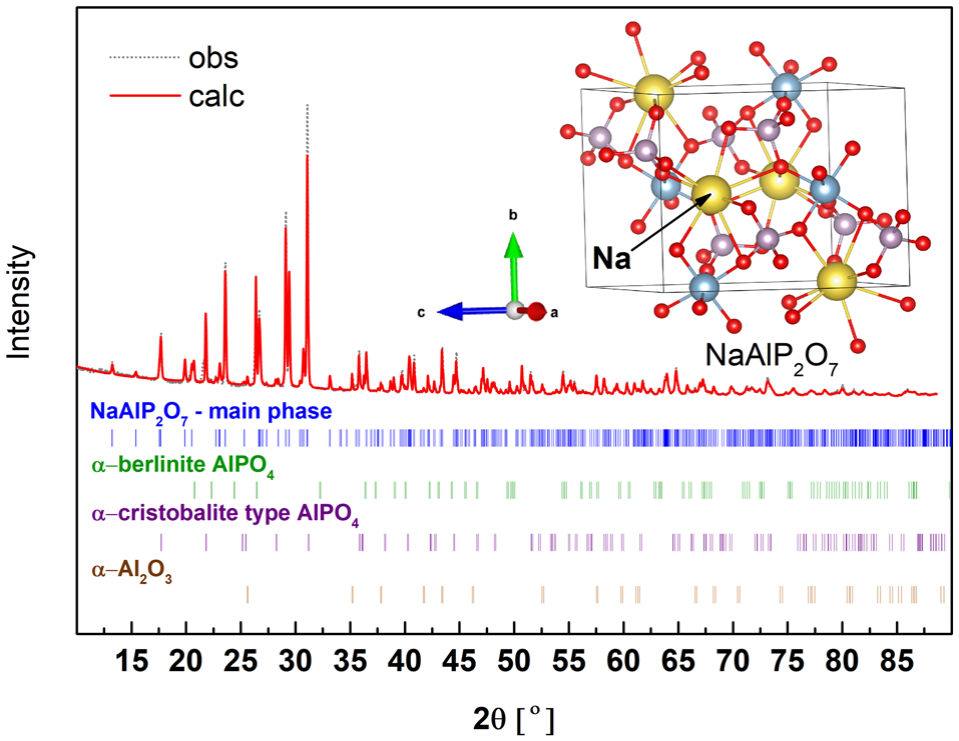
X-ray diffraction pattern (obs) and fit (calc) of NaAlP_2_O_7_. Inset, the unit cell of the compound with the optimized atom positions.

In Al_2_O_3_-P_2_O_5_ there is no known pure compound containing Q^1^ structural units. Therefore, sodium-containing NaAlP_2_O_7_ was chosen where the unit cell is built of Q^1^–Q^1^ dimers. The XRD pattern of the synthesized material is presented in Fig. 12. The main crystal phase present in the obtained material is NaAlP_2_O_7_ (c.a. 85 wt%). Secondary minor phases are AlPO_4_ of the berlinite and cristobalite type and Al_2_O_3_. The detailed phase composition is given in Table S2.3 (suppl.). The main phase crystallizes in a monoclinic P12_1_/c1 space group and the fitted crystal structure parameters are *a* = 7.197(4) Å, *b* = 7.704(5) Å, *c* = 9.314(5) Å, β = 111.72(5)^o^.

In the unit cell Q^1^-Q^1^ (P_2_O_7_) dimers are connected to [AlO_6_] octahedra and sodium polyhedra. In this case, the distance of P-O_B_ is in the range of 1.612–1.616 Å and P-O_NB_ in the range of 1.499–1.527 Å. The excess of the P positive charge is now redistributed over 3 non-bridging oxygens in the Q^1^ unit. Because the unit has only one bond longer (P–O_B_) and three of similar lengths (P-O_NB_), the idealized symmetry of the unit is the same as the Q^3^ unit. Thus, Q^1^ has the same C_3v_ point group as Q^3^.

**Figure 13:**
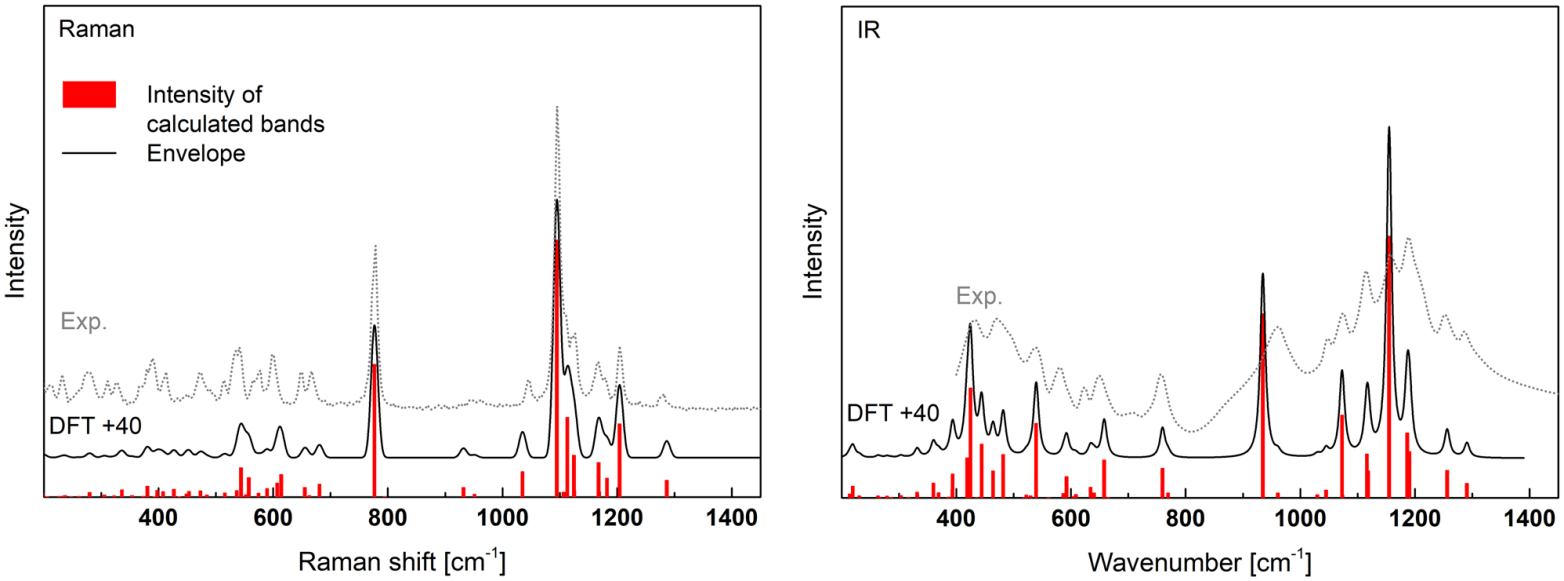
Calculated Raman and IR spectra of NaAlP_2_O_7_ shifted by + 40 cm^−1^ and experimental Raman and IR spectra (Exp.) of the synthesized material.

The calculated and measured Raman and IR spectra are presented in Fig. 13. Good agreement is also observed between the theory and the experiment. The best results may be obtained after including about + 40 cm^−1^ shift of the theoretical spectra. The detailed positions and intensity of the calculated active bands are summarized in the supp. (Table S1.5), and in the simplified form in Table 5.

**Table 5:** The calculated Raman and IR active modes of NaAlP_2_O_7_ (more details in the supplementary material Table S1.5).

Frequency (> 200) [cm^−1^]	Intensity Raman (the most intense frequencies [cm^−1^])	Intensity IR (the most intense frequencies [cm^−1^])	Assignment to Q^i^ idealized vibrations and P–O_B_–P
< 330	Very weak	Very weak	Lattice vibrations and librations
341–641	Weak (341, 504 (s), 518, 567, 574 and 641)	Medium (354, 379, 385 (s), 404, 424, 442, 499, 552 and 618)	Bending (A_1_) in P-O_B_-P and Asymmetric (E) deformation of 3(P-O_NB_) in Q^1^ (in [AlO_6_] and Na^+^ environment)
720–755	Strong (737 (s))	Weak (720 (s))	Symmetric stretching (A_1_) in P-O_B_-P and symmetric deformation (A1) of 3(P-O_NB_) in Q^1^ (in the [AlO_6_] and Na^+^ environment)
892–921	Very weak (895 (s))	Strong	Asymmetric stretching (B_1_) in P-O_B_-P (in [AlO_6_] and Na^+^ environment)
990–1067	Very strong (1055 (s))	Medium (1033 (s))	Symmetric stretching (A_1_) of 3(P-O_NB_) in Q^1^ (in [AlO_6_] and Na^+^ environment)
1073–1251	Medium (1073 (s), 1085, 1128, 1142, 1164 and 1247)	Strong (1076, 1115 (s), 1147, 1151 and 1216)	Asymmetric stretching (E) of 3(P-O_NB_) in Q^1^ (in [AlO_6_] and Na^+^ environment)

s—the strongest in range.

The most intense band in the Raman spectrum is at 1055 cm^−1^ related to the symmetric stretching (A_1_) modes of 3(P–O_NB_) in Q^1^. With this feature are associated bands of higher frequencies in the range of 1073–1251 cm^-1^ related to asymmetric stretching modes (E) of 3(P-O_NB_) in Q^1^. However, the intensity of the asymmetric vibrations is considerably lower. The second strong band is at 737 cm^-1^ due to symmetric stretching (A_1_) in P–O_B_–P and symmetric deformation (A1) of 3(P–O_NB_).

The IR spectrum is characterized by two strong groups of bands. The first in the range of 892–921 cm^−1^ is related to asymmetric stretching vibrations (B_1_) of P–O_B_–P. The second in the range of 1073–1251 cm^−1^ is related to the asymmetric stretching modes (E) of 3(P–O_NB_) in Q^1^. The medium strength has bands related to bending (A_1_) in P–O_B_–P, asymmetric deformation (E) of 3(P-O_NB_), and symmetric stretching (A_1_) of 3(P-O_NB_) (see Table 5).

### α-Cristobalite type AlPO_4_

**Figure 14:**
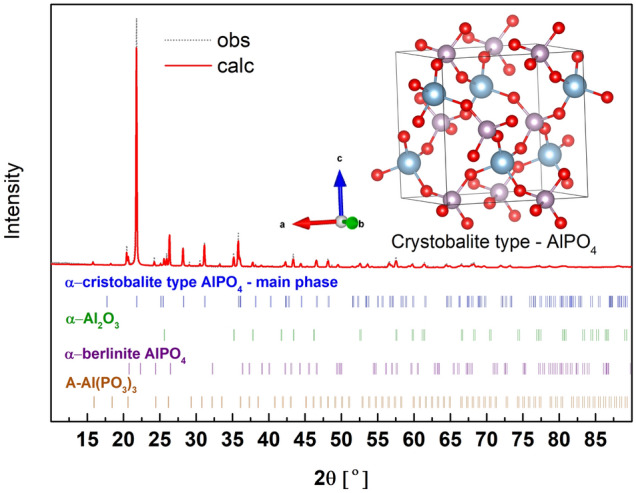
X-ray diffraction pattern (obs) and the fitted (calc) of α-cristobalite type AlPO_4_. Inset, the unit cell of the compound with the optimized atom positions.

The powder XRD diffraction pattern of the cristobalite type of AlPO_4_ is presented in Fig. 14. In this case, we were unable to obtain the pure phase. The main crystalline compound was assumed to be AlPO_4_ (c.a. 67 wt%). The rest of the crystalline phases in the sample are Al_2_O_3_ (c.a. 16 wt%) with the minor addition of A-Al(PO_3_)_3_ and the berlinite type of AlPO_4_. The detailed phase composition and the quantified analysis are summarized in Table S2.4 (supp.). The main phase crystallizes in a orthorombic C222_1_ space group and the fitted crystal structure parameters are *a* = 7.103(4) Å, *b* = 7.096(3) Å, *c* = 7.011(5) Å.

The α-cristobalite type AlPO_4_ is built of Q^0^ structural units connected by [AlO_4_] tetrahedra. In the crystal structure, there are no bridging oxygen atoms, and all the oxygens are non-bridging. The length of the P-O_NB_ bond is in the range of 1.521–1.523 Å. Due to the fact that all oxygens in the [PO_4_] tetrahedrons have a similar P-O distance, the tetrahedron is close to ideal and can be described by symmetry of the T_d_ point group.

**Figure 15:**
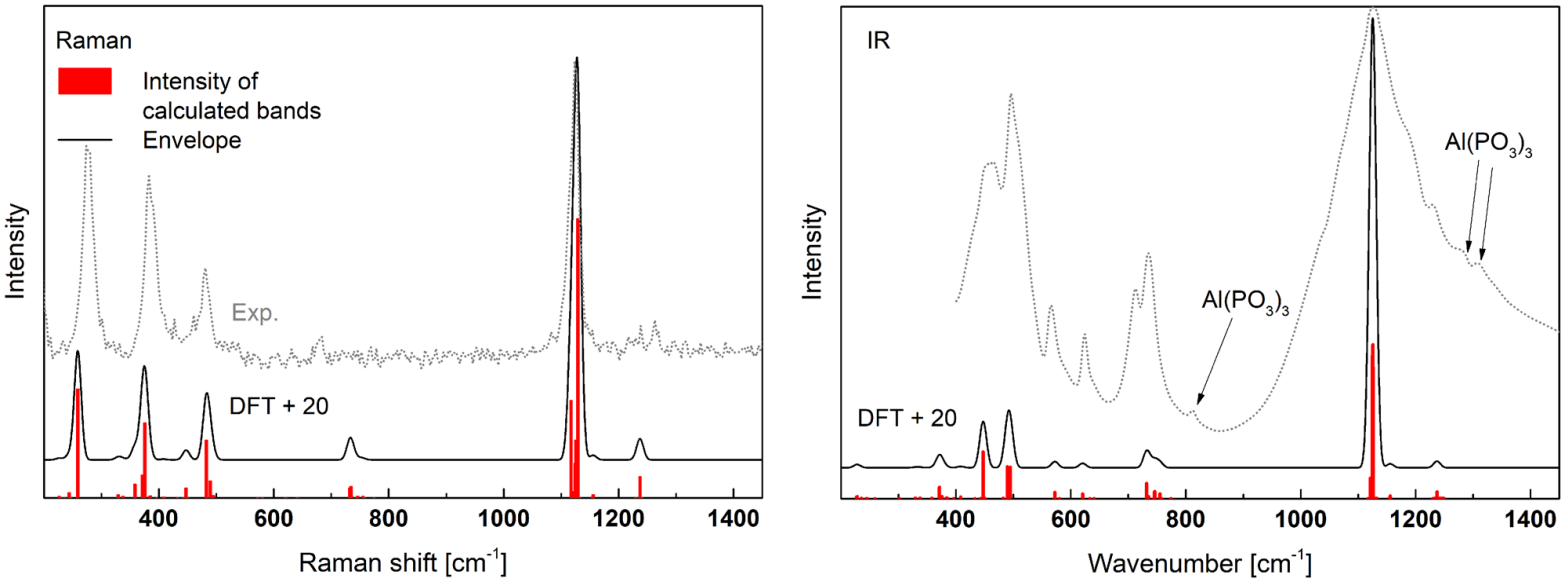
Calculated Raman and IR spectra of α-cristobalite type AlPO_4_ shifted by + 20 cm^-1^ and experimental IR and Raman spectra (Exp.) of the synthesized material.

The calculated and experimental Raman and IR spectra of the samples are presented in Fig. 15. The material obtained is polyphase in the case where characteristic vibrations of A-Al(PO_3_)_3_ were also detected in the IR spectra. On the other hand, Raman spectroscopy is measured at a point, and it was possible to detect the spectrum of the pure cristobalite phase. In this case, good agreement between theory and experiment can also be evidenced. The best results were obtained when the calculated spectra had been shifted to a value of + 20 cm^−1^. A detailed description of the active modes is given in Table S1.6 (supp.) and the simplified version in Table 6.

**Table 6:** The calculated Raman and IR active modes of α-cristobalite type AlPO_4_ (more details in the supplementary materials Table S1.6).

Frequency (> 200) [cm^−1^]	Intensity Raman (the most intense frequencies [cm^−1^])	Intensity IR (the most intense frequencies [cm^−1^])	Assignment to Q^i^ idealized vibrations and P–O_B_–P
< 239	Medium (239 (s))	Very weak	Lattice vibrations and librations
309–365	Medium (356 (s))	Medium (351 (s))	Symmetric bending (E) of Q^0^ (in [AlO_4_] environment)
476–735	Medium (462 (s))	Weak (428 (s), 470 and 475)	Asymmetric deformation (F_2_) of Q^0^ (in [AlO_4_] environment)
1097–1106	Medium (1097 (s) and 1106)	Very strong (1102 and 1106 (s))	Asymmetric stretching (F_2_) of Q^0^ (in [AlO_4_] environment)
1109	Very strong	Very weak	Symmetric stretching (A_1_) of Q^0^ (in [AlO_4_] environment)
1112–1217	Very weak (1217 (s))	Very weak (1217 (s))	Asymmetric stretching (F_2_) of Q^0^ (in [AlO_4_] environment)

S—the strongest in range.

The Raman spectrum is characterized by a strong band at 1109 cm^−1^ due to symmetric stretching vibrations (A_1_) in Q^0^. In the spectrum there are also visible 3 characteristic bands in the range of 239–735 cm^-1^. The two in the range of 325–735 cm^−1^ are related to the symmetric bending (E) and asymmetric deformation (F_2_) modes of Q^0^. The band 239 cm^−1^ is due to lattice vibrations.

The most characteristic feature of the IR spectrum is a strong band at 1106 cm^−1^ that may be assigned to asymmetric stretching modes (F_2_) in Q^0^. Also, in the IR spectra there are good visible medium vibrations related to symmetric bending (E) of Q^0^ and weak vibrations related to asymmetric deformation (F_2_) of Q^0^.

### α-Berlinite AlPO_4_

**Figure 16:**
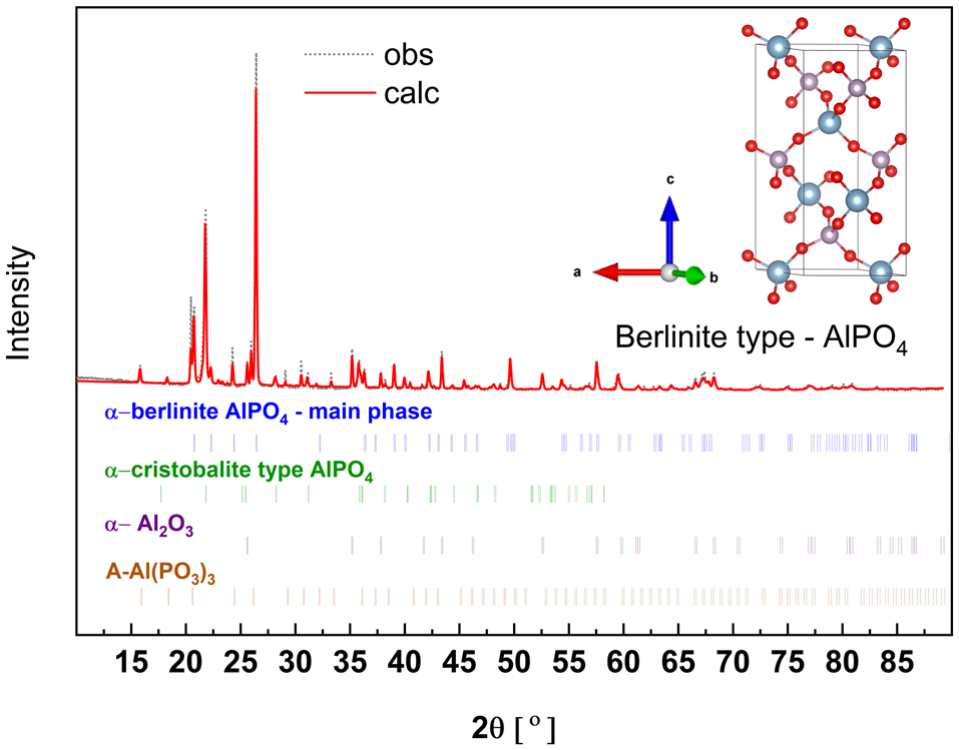
X-ray diffraction pattern (obs) and the fitted (calc) of α-berlinite type AlPO_4_. Inset, the unit cell of the compound with the optimized atom positions.

The next polymorphic form of AlPO_4_ is the berlinite type. The XRD pattern of the synthesized material is given in Fig. 16. As can be seen, the synthesized material was polyphase. The main crystalline compound is the assumed berlinite type of AlPO_4_ (c.a. 57 wt%). There are also Al_2_O_3_, A-Al(PO_3_)_3,_ and other polymorphic phase of AlPO_4_ such as cristobalite. The detailed phase composition of the synthesized sample is summarized in Table S2.5 (supp.). The main phase crystallizes in a trigonal P3_2_21 space group and the fitted crystal structure parameters are a = b = 4.948(5) Å, c = 10.950(7) Å.

The crystal structure of berlinite is very similar to that of cristobalite AlPO_4_. The unit cell is composed of Q^0^ structural units connected by [AlO_4_] tetrahedra with the P-O_B_ distance in the range of 1.507–1.512 Å.

**Figure 17:**
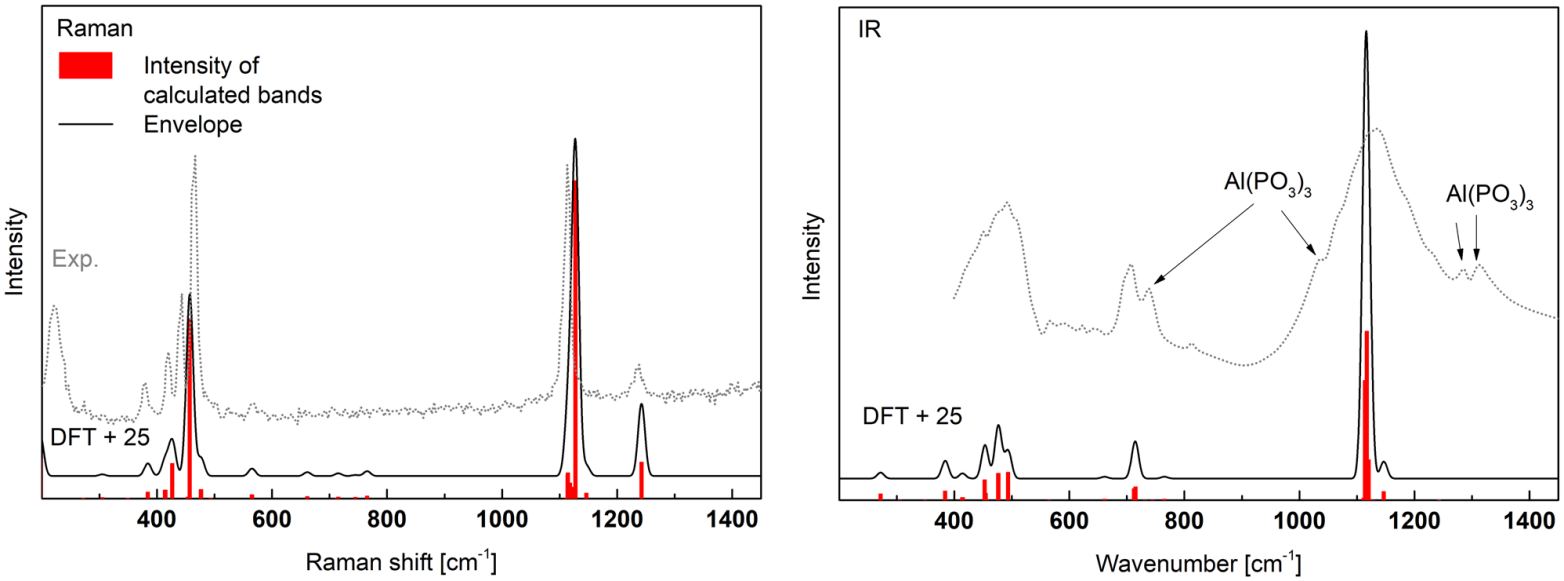
Calculated Raman and IR spectra of α-berlinite type AlPO_4_ shifted by + 20 cm^−1^ and experimental IR and Raman spectra (Exp.) of the synthesized material.

The calculated and experimental Raman and IR spectra are shown in Fig. 17. In this case, the best agreement is obtained for the theoretical data shifted by + 25 cm^−1^. Similarly to the above, the most suitable Raman spectrum was chosen to compare with the calculated spectra. In the IR spectrum, in addition to bands related to α-cristobalite type AlPO_4_ there are weak bands of A-Al(PO_3_)_3_. The detailed Raman and IR active modes with the proper assignment are given in Table S1.7 (supp.) and the simplified in Table 7.

**Table 7:** The calculated Raman and IR active modes of α-berlinite type AlPO_4_ (more details in the supplementary materials Table S1.7).

Frequency (> 200) [cm^−1^]	Intensity Raman (the most intense frequencies [cm^−1^])	Intensity IR (the most intense frequencies [cm^−1^])	Assignment to Q^i^ idealized vibrations and P–O_B_–P
< 280	Very weak	Very weak	Lattice vibrations and librations
325–432	Medium (389, 402 and 432 (s))	Weak (428 (s))	Symmetric bending (E) of Q^0^ (in [AlO_4_] environment)
451–741	Very weak	Medium (451 (s), 469 and 690)	Asymmetric deformation (F_2_) of Q^0^ (in [AlO_4_] environment)
1087–1095	Weak (1089 (s))	Very strong (1089, 1092 (s) and 1095)	Asymmetric stretching (F_2_) of Q^0^ (in [AlO_4_] environment)
1103–1122	Very strong (1103 (s))		Symmetric stretching (A_1_) of Q^0^ (in [AlO_4_] environment)
1217	Weak	Very weak	Asymmetric stretching (F_2_) of Q^0^ (in [AlO_4_] environment)

S—the strongest in range.

On the Raman spectrum, the strongest band at 1109 cm^−1^ may be assigned to vibrations of symmetric stretching (A1) in Q^0^. The position of this band is very similar in α-cristobalite type AlPO_4_ and α-berlinite. The medium bands are present in the range of 451–741 cm^−1^ and are related to the symmetric bending modes (E) of Q^0^. The bands related to the asymmetric deformation (F_2_) of Q^0^ are very weak in the α-berlinite spectrum.

On the IR spectrum, the strongest band is at 1092 cm^−1^ due to asymmetric stretching vibrations (F_2_) in Q^0^. Furthermore, bands in the range of 451–741 cm^−1^ related to asymmetric deformation (F_2_) of Q^0^ are clearly visible in the spectrum.

## Discussion

Analyzing the obtained experimental and theoretical results one may see that the main intense bands are due to P-O bonds vibrations in Q^i^ structural units in the higher frequency range and P-O-P in the midregion. This is the most well seen in the case of the Raman spectra, wherein in the most considered cases the two bands are dominating. The intensity of the midband decreases with the Q^i^ index, which is related to the decrease of the number of P–O–P linkages. The position of the symmetric modes is centered at frequencies lower than asymmetric.

**Figure 18:**
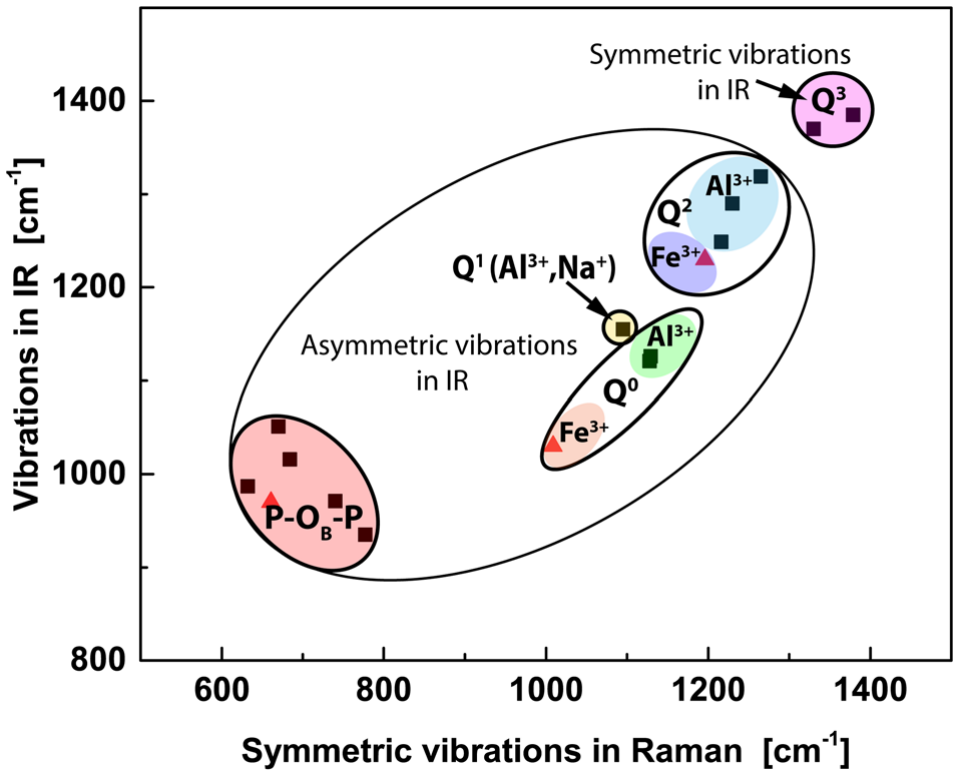
The most intense bands positions of symmetric and asymmetric stretching vibrations of P-O_NB_ in Q^i^ and P-O_B_-P in Raman and IR spectra. Al^3+^- vibrations in aluminum phosphates, (Al^3+^,Na^+^) in NaAl(P_2_O_7_) and Fe^3+^ in iron phosphates. Data in red triangles from^42–44^.

The position of the bands related to Q^i^ units depends on the value of the parameter i, and with the parameter increase the position shifts towards higher values, which is presented in Fig. 18. The separated ranges of the vibrations for specific Q^i^ spices can be distinguished. This shows that Raman and IR spectroscopies may provide important information concerning the Q^i^ distribution in materials.

On the other hand, the modes related to the different vibrations in [AlO_x_] polyhedrons are very weak, and it seems that spectroscopies cannot be utilized to distinguish the Al-O environment. However, it should be notated that the occurrence of polyhedrons influence the position of the vibrations of Q^i^ units as shown in Fig. 18. Comparing the results with the data summarized in^42–44^ with respect to iron phosphates, it can be detected that Al^3+^ shifts the Q^i^ vibration toward higher frequencies compared to Fe^3+^. This may be useful in the case of materials containing iron and aluminum to differentiate Q^i^ species connected with Al^3+^ and Fe^3+^ cations as in glasses. The position of the band related to the symmetric stretching modes of 3(P-O_NB_) in the structural unit of NaAlP_2_O_7_ is very close to the band in Q^0^ (α-cristobalite or α-berlinite). The position of this band for Q^1^ is usually higher than Q0 [19, 48]. The ionic nature of Na^+^ shifts the band toward lower values. A similar effect can be detected for Fe and Al, and iron, which is more ionic to oxygen than aluminum, also lowers the position of the band in Q^i^ species^19,42,45,46^.

**Figure 19:**
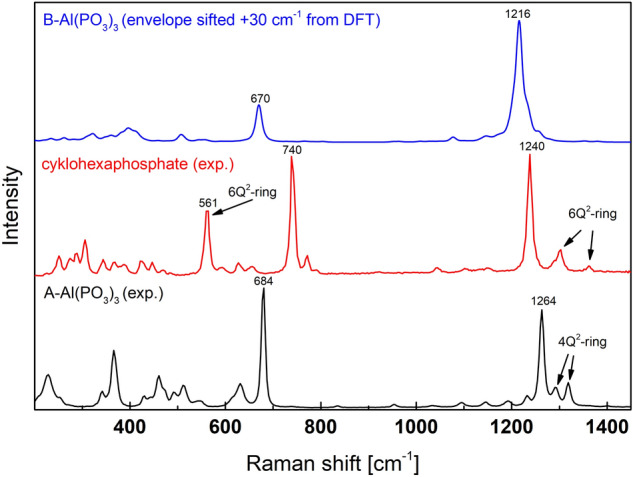
Comparison of experimental spectra of Al(PO_3_)_3_ polymorphs and envelope of calculated spectra of B-Al(PO_3_)_3_. The characteristic bands for the 6Q^2^-ring and the 4Q^2^-ring were marked in the figure.

Another important observation is evidence of mid-intensity bands characteristic of phosphate rings vibrations in Al(PO_3_)_3_. The vibrations are located at higher frequencies next to the most intense band (Fig. 19). The bands characteristic to 6Q^2^- and 4Q^2^-rings are well visible and allow to distinguish between different Al(PO_3_)_3_ polymorphic forms.

Additionally, the main intense band for the ring structures is shifted toward the higher values, and the shift is the highest for the 4Q^2^-rings. The shift is probably related to the increase in the stiffness of bonds in ring structures. The rings are more rigid than the chains, and the smaller rings are more rigid than the larger ones. Therefore, the position moves to a higher frequency.

## Conclusions

Theoretical Ramana and IR spectra of aluminum phosphate compounds containing Q^i^ structural units were calculated from Q^0^ to Q^3^ and characteristic vibration modes were described. The selected compounds were synthesized, and the experimental spectra were compared with those of theoretical. It was evidenced by the good agreement between the theoretical and experimental results. The best convergence was obtained when the calculated Ramana and IR spectra were shifted in the range of + 20–+ 40 cm^−1^ without applying any scaling factor.

It was evidenced that the Raman spectra are characterized by the presence of two characteristic bands in the mid-and high-frequency ranges. The mid-band is originating from P–O–P bridges, whereas the higher band is the result of P–O vibrations in Q^i^ tetrahedrons. The position of the high-frequency band is correlated with the index i in the Q^i^ species and can be used to predict the distribution of Q^i^ units in materials.

In the case of the Raman spectra, symmetric vibrations are much more intense than asymmetric, whereas in the case of the IR the opposite effect is evidenced. The IR spectra are also dominated by two bands because of the vibrations of P-O-P and P-O in Q^i^ units, similar to Raman.

For Al(PO_3_)_3_ and AlPO_4_ differences in Raman spectra related to different polymorphic forms were observed and described.

## Materials and methods

### Simulations

Calculations of Raman and IR spectra were conducted for the crystalline compounds presented in Table 8 using Quantum Espresso 6.4 software^47^. In the calculation procedure, the unit cell parameter was taken from the reference, and the positions of the atoms were optimized. The unit cell parameters were not optimized to decrease the calculation time, especially for the big unit cells. This approach may limit the accuracy of the results. Nevertheless, most of the predicted spectra are compared to the experimental or literature data to validate the calculation procedure.

**Table 8:** Calculated and synthesized crystalline compounds.

Crystalline compound	Crystal system	Symmetry space group name in Hermann–Mauguin notation	Phosphate structural unit in crystalline compound (Q^i^ notation)	Unit cell parameters: a, b, c [Å] α, β, γ [^o^]	Source, COD database ID
o’-P_2_O_5_	Orthorhombic	P n m a	Q^3^	9.193, 4.890, 7.162 *α* = *β* = *γ* = 90	^25^, 2,003,536
B-Al(PO_3_)_3_	Monoclinic	I 1 c 1	Q^2^ (chains)	10.423, 18.687, 9.222 *α* = *γ* = 90, *β* = 81.630	^26^, 2,106,619
A-Al(PO_3_)_3_	Cubic	I $\overline{4 }$ 3 d	Q^2^ (4 membered rings)	a = b = c = 13.63 *α* = *β* = *γ* = 90	^27^, 1,010,266
Aluminum cyclohexaphosphate-Al(PO_3_)_3_	Monoclinic	P 1 2_1_/c 1	Q^2^ (6 membered rings)	6.093, 15.068, 8.202 *α* = *γ* = 90, *β* = 105.166	^28^, 2,225,399
NaAl(P_2_O_7_)	Monoclinic	P 1 2_1_/c 1	Q^1^	7.203, 7.710, 9.326 *α* = *γ* = 90, *β* = 111.743	^48^, 8,103,838
α-Cristobalite type AlPO_4_	Orthorhombic	C 2 2 2_1_	Q^0^	7.084, 7.082, 6.999 *α* = *β* = *γ* = 90	^31^, 1,532,548
α-Berlinite (AlPO_4_)	Trigonal	P 3_2_ 2 1	Q^0^	a = b = 4.944, c = 10.950 *α* = *β* = 90, *γ* = 120	^33^, 9,006,404

The PWscf program included in the Quantum Espresso package was used to optimize positions and perform self-consistent field SCF calculations. This program is based on Density Functional Theory (DFT), a plane-wave basis set, and pseudopotentials. The local density approximation LDA and optimized norm-conserving Vanderbilt scalar relativistic pseudopotentials from the Pseudo Dojo project^49,50^ were used in the calculations. The cut-off energy for valence electrons plane-waves basis set and charge densities were 50 and 200 Ry, respectively. The Monkhorst–Pack k-point sampling scheme with a 3 × 3 × 3 mesh grid was used. Self-consistency and convergence of total energy for ionic minimization were set to 10^–8^ and 10^–4^ Ry, respectively. The results of the SCF calculations for optimized structures of crystalline compounds were used in Raman and IR spectra calculations. The calculations of Raman and IR spectra were performed using the PHonon program from the Quantum Espresso package which is based on density functional perturbation theory (DFPT). The k-point grid remained the same as in the previous calculations. The threshold for self-consistency was set at 10^–12^ Ry. The selected k-point mesh was sufficient to obtain satisfactory results and at the same time a decent calculation time. To better visualize IR and Raman theoretical spectrum, the envelopes were calculated by a script written in Python using SciPy library^51^.

### Synthesis

Crystalline compounds included in Table 8 were synthesized, except o’-P_2_O_5_ and B-Al(PO_3_)_3_. Stoichiometric quantities of chemically pure NH_4_H_2_PO_4_, Al_2_O_3_, and Na_2_CO_3_ were used. The synthesis was conducted according to the following procedure. The starting NH_4_H_2_PO_4_ was decomposed into H_3_PO_4_ by heating to 200 °C in a Al_2_O_3_ crucible in an electric furnace. The H_3_PO_4_ obtained was kept at 200 °C for 2 h. The molten H_3_PO_4_ was thoroughly mixed with Al_2_O_3_ or/and Na_2_CO_3_. The resulting pastes were placed in an alumina combustion boat. The samples were sintered according to the temperatures in Table 9. Synthesis temperatures were selected according to^4,11,28^. Due to the high hygroscopicity of P_2_O_5,_ it must be synthesized in tightly closed containers. Also, the measurement procedure using XRD, Raman, or IR spectroscopy must be performed in the absence of air^25^. The synthesis of o’-P_2_O_5_ has been ongoing for several weeks. Due to these difficulties, it was decided to abandon the synthesis of o’-P_2_O_5_. B-Al(PO_3_)_3_ was not obtained from molten H_3_PO_4_ and Al_2_O_3_ at temperatures of 550 and 900 °C. A synthesis at 700 °C was also performed and a small amount of B-Al(PO_3_)_3_ is present in the sample but not enough to compare with the calculated spectra. This sample contains mainly A-Al(PO_3_)_3_ (Raman and IR spectra in the supplementary materials Fig. S2.1 and Fig. S2.2). Obtaining B-Al(PO_3_)_3_ from melting Al_2_O_3_ with HPO_3_ has been reported^26^. Also, in^30^ V. Bemmer et al. report only aluminum cyclohexaphosphate or/and A-Al(PO_3_)_3_ obtained from H_3_PO_4_ with various precursors (Al(OH)_3_, Al(NO_3_)_3_ or AlCl_3_) water solutions at temperatures 500 °C and 800 °C.

**Table 9:** Synthesis temperatures of crystalline compounds.

Expected crystalline compound at room temperature:	Synthesis temperature [°C]
A-Al(PO_3_)_3_	900
Aluminum cyclohexaphosphate Al(PO_3_)_3_	550
NaAlP_2_O_7_	850
α-Cristobalite type AlPO_4_	1050
α-Berlinite (AlPO_4_)	750

All of the steps of the synthesis were performed in an air atmosphere. The samples were gradually heated to the synthesis temperature for 5 h and then kept at the temperature for 8 h. Then were cooled to room temperature with the furnace. The obtained materials were visibly porous as a result of the release of water vapor during synthesis. The samples were then removed from the containers and crushed into smaller pieces. After berlinite synthesis, the part of Al_2_O_3_ did not react. Therefore, the sample was ground in an agate mortar and powder was pressed into a tablet using a hydraulic press. The pressed sample was sintered at 750 °C for 8 days.

The crystalline compositions of the samples were checked using XRD. Powder XRD measurements were carried out with a Philips X’Pert Pro diffractometer and Cu K_α1_ radiation. The phase compositions of the obtained materials and the crystal structure parameters have been obtained using the Rietveld method using GSAS-II software^52^.

All Raman measurements were made using a Witec Alpha 300 M + Confocal Raman Imaging system with the application of a 50 × air objective (Zeiss, LD EC Epiplan-Neofluar, NA = 0.55). The spectrometer was equipped with an air-cooled solid-state laser operating at 488 nm, a CCD detector that was cooled to − 60 °C, and 600 grooves per mm of gratings. Raman spectra of each sample were collected with two scans and an integration time of 20 s.

Spectroscopic studies were carried out in middle infrared (MIR) regions (4000–400 cm^−1^) using a Fourier transformation spectrometer (FT-IR). Samples were prepared using tablet methods in KBr. Measurements were collected after 128 scans at a resolution of 4 cm^−1^.

## Supplementary Information

Supplementary Information.

## Data Availability

The datasets generated during and/or analysed during the current study are available from the corresponding author on reasonable request.
