# Supplementary material for: Vibrational characteristics of aluminum–phosphate compounds by an experimental and theoretical approach
Source: Sci Rep. 2022 Oct 19;12:17495. doi: 10.1038/s41598-022-22432-5 (PMC9581984; doi:10.1038/s41598-022-22432-5)
Supplement: Supplementary file 1 — Supplementary Information. [file 41598_2022_22432_MOESM1_ESM.docx]

**Supplementary Materials for**

**Vibrational characteristics of aluminum–phosphate compounds by an experimental and theoretical approach**

**Table S1.1.** Calculated Raman and IR vibrations of **o’-P_2_O_5_** (vibrations < 0.01 and nonactive in Raman and IR have been omitted).

| Frequency  (>200)  [cm^-1^] | Intensity Raman | Intensity IR | Assignment to Q^i^ idealized vibrations and P-O_B_-P | Vibrations in crystal (point group D_2h_) |
| --- | --- | --- | --- | --- |
| 243.49 | 11.8431 | Non | Lattice vibrations and Librations | B_2g_ |
| 265.93 | 22.2853 | Non |  | B_1g_ |
| 266.90 | Non | 1.9364 |  | B_1u_ |
| 270.34 | Non | 0.6917 |  | B_3u_ |
| 283.77 | Non | 0.0385 |  | B_2u_ |
| 289.00 | 0.7362 | Non |  | B_3g_ |
| 289.52 | 26.39 | Non |  | A_g_ |
| 298.36 | 1.4215 | Non |  | B_2g_ |
| 304.56 | 2.9542 | Non |  | B_1g_ |
| 368.29 | Non | 21.7747 | Asymmetric deformation (E) of 3(P-O_B_) in Q^3^ | B_1u_ |
| 381.38 | 8.3956 | Non |  | A_g_ |
| 390.83 | Non | 0.087 |  | B_2u_ |
| 395.10 | 1.0234 | Non |  | B_3g_ |
| 395.37 | Non | 67.4877 |  | B_3u_ |
| 412.07 | 4.5379 | Non |  | B_1g_ |
| 423.65 | 2.9197 | Non |  | A_g_ |
| 426.01 | 3.3117 | Non |  | B_3g_ |
| 431.17 | 1.3143 | Non |  | B_2g_ |
| 435.96 | Non | 10.3573 |  | B_2u_ |
| 438.22 | Non | 10.5052 |  | B_1u_ |
| 456.63 | Non | 0.424 |  | B_3u_ |
| 468.61 | 6.1009 | Non |  | B_1g_ |
| 482.59 | 0.2401 | Non |  | B_2g_ |
| 500.85 | Non | 8.1611 | Symmetric (A_1_) and Asymmetric deformation (E) of 3(P-O_B_) in Q^3^ in different positions | B_1u_ |
| 504.76 | Non | 12.1731 | Bending (A_1_) in P-O_B_-P | B_2u_ |
| 506.86 | 1.1003 | Non | Symmetric (A_1_) and Asymmetric deformation (E) of 3(P-O_B_) in Q^3^ in different positions | A_g_ |
| 537.54 | 0.0022 | Non | Asymmetric deformation (E) of 3(P-O_B_) in Q^3^ | B_3g_ |
| 601.89 | 342.7218 | Non | Symmetric (A_1_) and Asymmetric deformation (E) of 3(P-O_B_) in Q^3^ in different positions | A_g_ |
| 602.97 | Non | 14.3392 | Asymmetric deformation (E) of 3(P-O_B_) in Q^3^ | B_1u_ |
| 629.33 | Non | 6.3477 |  | B_2u_ |
| 632.50 | 0.0136 | Non |  | B_3g_ |
| 733.64 | Non | 1.3594 | Symmetric stretching (A_1_) in P-O_B_-P | B_2u_ |
| 746.41 | 1.2048 | Non |  | B_3g_ |
| 767.92 | 25.0872 | Non |  | A_g_ |
| 776.04 | Non | 0.1357 |  | B_3u_ |
| 777.66 | 14.7519 | Non |  | B_2g_ |
| 777.78 | Non | 3.3494 |  | B_1u_ |
| 780.88 | 12.4477 | Non |  | B_1g_ |
| 935.95 | 0.8125 | Non | Asymmetric stretching (B_1_) in P-O_B_-P | B_1g_ |
| 936.67 | Non | 168.149 |  | B_3u_ |
| 939.21 | 2.53 | Non |  | B_3g_ |
| 954.63 | 12.6923 | Non | Asymmetric stretching (E) of 3(P-O) in Q^3^ | A_g_ |
| 957.01 | Non | 113.774 |  | B_1u_ |
| 1098.87 | Non | 51.9711 | Symmetric (A_1_) and Asymmetric (E) stretching of 3(P-O_B_) in Q^3^ in different positions | B_2u_ |
| 1112.66 | 0.326 | Non | Asymmetric stretching (B_1_) in P-O_B_-P | B_3g_ |
| 1119.37 | 3.258 | Non | Symmetric stretching (A_1_) of 3(P-O_B_) in Q^3^ | A_g_ |
| 1124.06 | Non | 0.328 | Symmetric stretching (A_1_) and Asymmetric stretching (E) of 3(P-O_B_) in Q^3^ in different positions | B_1u_ |
| 1143.61 | Non | 0.0543 |  | B_2u_ |
| 1149.6 | 1.937 | Non | Asymmetric stretching (E) of 3(P-O_B_) in Q^3^ | B_3g_ |
| 1281.67 | Non | 44.0646 | Symmetric stretching (A_1_) of P=O_NB_ in Q^3^ | B_2u_ |
| 1299.88 | 203.1949 | Non |  | A_g_ |
| 1304 | 8.2544 | Non |  | B_3g_ |
| 1336.57 | Non | 2.2157 |  | B_1u_ |
| 1343.84 | 191.4451 | Non |  | A_g_ |
| 1348.54 | Non | 16.8702 |  | B_2u_ |
| 1355.04 | Non | 11.8556 |  | B_1u_ |
| 1376.1 | 8.2785 | Non |  | B_3g_ |

Non – nonactive, The most intense

In **o’-P_2_O_5_** only exists **Q^3^** structural units. This structural unit have one P=O_NB_ bound which is significantly shorter than three P-O_B_ bonds. Then the idealized (which has the same angles and lengths of P-O_B_ bonds) molecule of Q^3^ has C_3v_ symmetry. The vibrations of Q^3^ in P_2_O_5_ were assigned to the idealized molecule. The Fig. S.1.1 shows the vibrations occurring in the Q^3^ structural unit in **o’-P_2_O_5_** assigned to idealized Q^3^ which have $\Gamma_{\mathrm{osc}}=3A_{1}+3E$ without translation and rotation.


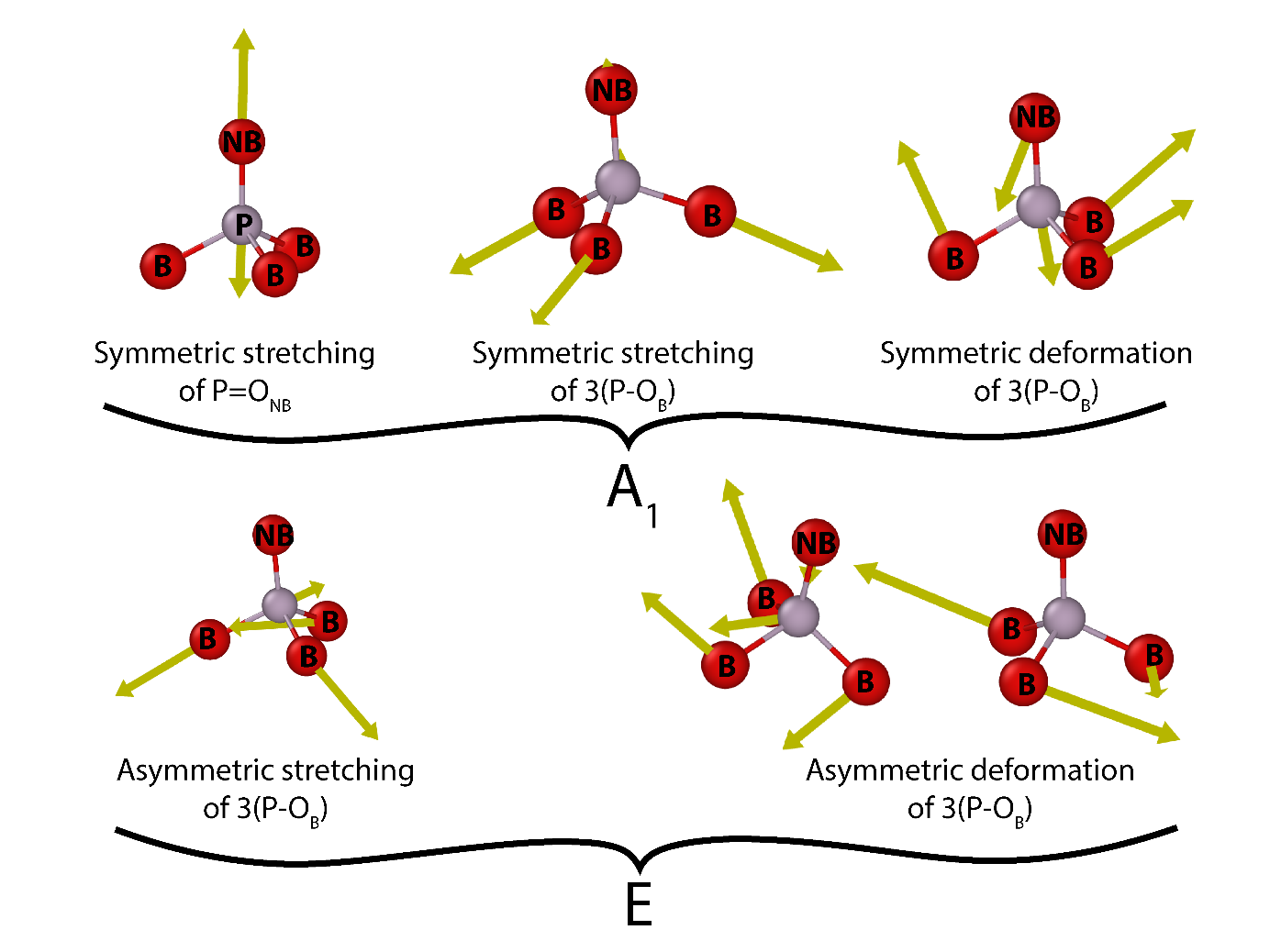


Fig. S1.1. Examples of vibrations in **o’-P_2_O_5_** assignment to vibrations of idealized Q^3^ unit.

Similarly to Q^3^, vibrations were assigned to idealized P-O_B_-P molecules, which are often used to describe vibrations in amorphous and crystalline phosphate materials. Idealized P-O_B_-P molecule has C_2v_ symmetry and $\Gamma_{osc}=2A_{1}+B_{1}$ without translation and rotation (Fig. S.1.2).


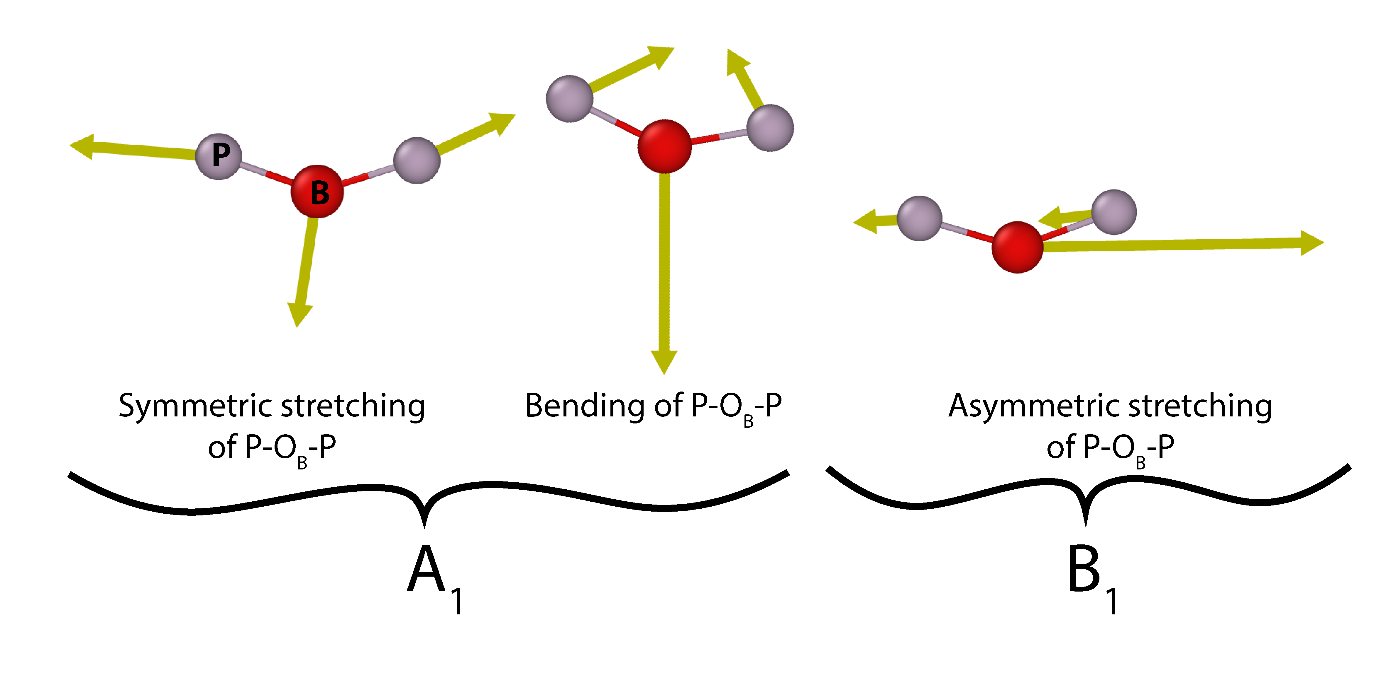
Fig. S1.2. Examples of vibrations in **o’-P_2_O_5_** assignment to vibrations of idealized P-O_B_-P unit.

**Table S1.2.** Calculated Raman and IR vibrations of **B-Al(PO_3_)_3_** (vibrations < 0.01 and nonactive in Raman and IR have been omitted).

| Frequency (>200) [cm^-1^] | Intensity Raman | Intensity IR | Assignment to Q^i^ idealized vibrations and P-O_B_-P | Vibrations in crystal (point group C_s_) |
| --- | --- | --- | --- | --- |
| 201.39 | 1.6505 | 0.0485 | Lattice vibrations and Librations | A’’ |
| 204.61 | 32.0517 | 0.476 |  | A’ |
| 204.83 | 5.0707 | 0.0027 |  | A’’ |
| 213.51 | 0.1976 | 0.7459 |  |  |
| 218.36 | 4.7264 | 0.4083 |  | A’ |
| 222 | 4.2658 | 0.0035 |  | A’’ |
| 230.31 | 0.2202 | 0.0057 |  |  |
| 231.81 | 46.7996 | 0.2625 |  | A’ |
| 235.61 | 0.09 | 0.9591 |  | A’’ |
| 240.23 | 0.6688 | 0.984 |  | A’ |
| 248.08 | 1.5797 | 0.07 |  | A’’ |
| 250.35 | 15.3821 | 0.7862 | Asymmetric deformations (B_2_) in Q^2^ (Q^2^-chains in [AlO_6_] environment) | A’ |
| 254.24 | 2.135 | 0.0617 |  | A’’ |
| 255.46 | 5.5936 | 0.7951 |  | A’ |
| 257.63 | 0.9382 | 0.1033 |  | A’’ |
| 260.87 | 1.0533 | 0.4011 |  |  |
| 262.83 | 3.4536 | 0.1258 |  | A’ |
| 269.24 | 9.9308 | 1.6047 |  |  |
| 278.1 | 4.3689 | 0.0008 |  | A’’ |
| 279.53 | 28.7287 | 0.2461 |  | A’ |
| 283.33 | 15.2306 | 1.0707 |  |  |
| 283.99 | 0.8537 | 0.0522 |  | A’’ |
| 292.07 | 75.569 | 1.3931 |  | A’ |
| 292.82 | 1.3688 | 2.5793 |  | A’’ |
| 296.16 | 13.1473 | 0.9454 | Asymmetric deformations (B_2_) and twisting (A_2_) of 2(P-O_NB_) in Q^2^ (Q^2^-chains in [AlO_6_] environment) | A’ |
| 298.98 | 1.1839 | 2.6061 |  | A’’ |
| 301.67 | 0.1257 | 3.9324 |  |  |
| 305.13 | 5.6692 | 3.8833 |  | A’ |
| 314.12 | 0.0969 | 1.7945 |  | A’’ |
| 317.02 | 1.0484 | 0.7895 |  |  |
| 317.18 | 27.7004 | 3.1978 |  | A’ |
| 320.02 | 8.264 | 0.4424 |  |  |
| 323.67 | 0.8579 | 0.1718 |  | A’’ |
| 326.43 | 0.7793 | 0.0013 |  |  |
| 329.79 | 10.5766 | 0.4422 |  | A’ |
| 329.87 | 40.1947 | 1.651 |  |  |
| 333.92 | 3.109 | 12.5223 |  |  |
| 335.91 | 11.4397 | 0.543 |  | A’’ |
| 340.7 | 0.6088 | 0.9214 |  |  |
| 342.96 | 1.3192 | 0.168 |  | A’ |
| 347.51 | 14.2637 | 0.3619 |  |  |
| 349.85 | 25.3458 | 13.7338 |  |  |
| 352.33 | 38.6737 | 1.1272 |  | A’’ |
| 353.34 | 2.2013 | 7.6814 |  |  |
| 361.62 | 48.4033 | 18.8701 |  | A’ |
| 363.05 | 5.6553 | 1.4947 |  |  |
| 366.59 | 80.4398 | 0.2279 |  |  |
| 367.22 | 4.6299 | 0.9744 |  | A’’ |
| 369.36 | 0.3305 | 26.255 |  |  |
| 372.87 | 43.8903 | 21.6535 | Bending (A_1_) in P-O_B_-P and deformations (bending (A_1_) and asymmetric deformations (B_2_) of 2(P-O_NB_) in Q^2^ (Q^2^-chains in [AlO_6_] environment) | A’ |
| 378.17 | 7.1314 | 0.6231 |  | A’’ |
| 380 | 2.0205 | 0.4379 |  |  |
| 380.84 | 54.6163 | 13.1576 |  | A’ |
| 386.51 | 45.4328 | 28.7288 |  |  |
| 389.46 | 1.3859 | 29.4126 |  |  |
| 389.66 | 4.7484 | 7.1706 |  | A’’ |
| 394.26 | 0.965 | 10.5134 |  | A’ |
| 395.73 | 0.2928 | 2.1017 |  | A’’ |
| 396.57 | 5.6792 | 27.8374 |  | A’ |
| 398.42 | 5.3119 | 1.3627 |  | A’’ |
| 399.04 | 0.3777 | 0.1299 |  |  |
| 401.47 | 0.0214 | 19.8781 |  | A’ |
| 403.47 | 1.126 | 91.4259 |  | A’’ |
| 406.59 | 1.0773 | 7.0285 |  | A’ |
| 407.17 | 3.1924 | 7.9168 |  | A’’ |
| 412.95 | 0.3705 | 74.5792 |  | A’ |
| 415.39 | 0.6679 | 25.9138 |  | A’’ |
| 422.05 | 0.6333 | 35.9704 |  |  |
| 424.63 | 3.741 | 68.6967 |  |  |
| 430.2 | 5.1497 | 18.8826 |  |  |
| 430.66 | 4.0224 | 2.6492 |  | A’ |
| 434.7 | 0.132 | 29.152 |  | A’’ |
| 442.1 | 1.4318 | 27.0718 |  | A’ |
| 442.94 | 0.8478 | 2.1841 |  | A’’ |
| 443.83 | 1.4757 | 4.0994 |  | A’ |
| 448.02 | 0.5227 | 2.4709 |  | A’’ |
| 451.51 | 5.2419 | 0.2126 |  |  |
| 461.24 | 0.7922 | 186.0691 |  | A’ |
| 476.04 | 70.3094 | 0.0766 |  |  |
| 477.48 | 0.0155 | 0.0448 |  | A’’ |
| 480.42 | 30.0062 | 2.9092 |  | A’ |
| 486.27 | 7.5757 | 0.0011 |  | A’’ |
| 503.13 | 0.7344 | 3.5806 |  | A’ |
| 503.45 | 0.3037 | 0.5788 |  | A’’ |
| 507.92 | 0.7774 | 3.5769 |  |  |
| 512.4 | 12.1659 | 0.2248 |  | A’ |
| 513.53 | 4.2605 | 2.6731 |  |  |
| 514.41 | 0.7783 | 0.0016 |  | A’’ |
| 518.04 | 2.7092 | 0.5173 |  |  |
| 522.16 | 4.8705 | 0.1113 |  | A’ |
| 527 | 0.0005 | 14.799 |  | A’’ |
| 527.25 | 15.9749 | 0.6491 |  | A’ |
| 531.54 | 0.2375 | 5.7717 |  | A’’ |
| 533.09 | 7.2536 | 0.0314 |  | A’ |
| 544.84 | 0.6104 | 1.1603 |  |  |
| 548.82 | 0.2213 | 0.8219 |  | A’’ |
| 556.65 | 0.1154 | 15.8779 |  | A’ |
| 559.52 | 5.5653 | 0.6927 |  | A’’ |
| 565.55 | 1.2022 | 22.4986 |  | A’ |
| 568.63 | 1.2301 | 2.7801 |  | A’’ |
| 576.43 | 0.7173 | 0.0202 |  |  |
| 578.58 | 3.5169 | 33.9519 | Deformations (bending (A_1_) and asymmetric deformations (B_2_)) of 2(P-O_NB_) in Q^2^ (Q^2^-chains in [AlO_6_] environment) | A’ |
| 591.35 | 0.4955 | 5.0885 | Bending (A_1_) in P-O_B_-P and deformations (bending (A_1_) and asymmetric deformations (B_2_) of 2(P-O_NB_) in Q^2^ (Q^2^-chains in [AlO_6_] environment) |  |
| 592.87 | 0.8819 | 0.0031 |  | A’’ |
| 594.17 | 2.9084 | 6.3733 |  | A’ |
| 599.92 | 0.0127 | 2.7026 |  | A’’ |
| 608.77 | 6.1873 | 3.6659 | Bending (A_1_) in P-O_B_-P and asymmetric deformation (B_2_) of 2(P-O_NB_) in Q^2^ (Q^2^-chains in [AlO_6_] environment) | A’ |
| 609.29 | 0.7557 | 11.8588 |  |  |
| 609.88 | 0.1198 | 0.0903 |  | A’’ |
| 616.59 | 0.2236 | 0.0193 |  |  |
| 640.08 | 494.3093 | 0.0219 | Symmetric stretching (A_1_) in P-O_B_-P and bending (A_1_) of 2(P-O_NB_) in Q^2^  (Q^2^-chains in [AlO_6_] environment) | A’ |
| 663.4 | 0.0499 | 3.2136 |  |  |
| 666.78 | 0.1036 | 0.0489 |  | A’’ |
| 690.61 | 0.2036 | 0.9371 |  | A’ |
| 697.37 | 0.7671 | 4.0086 |  | A’’ |
| 699.68 | 2.9437 | 9.0289 |  |  |
| 709.84 | 0.0994 | 38.0146 |  |  |
| 710.98 | 1.759 | 0.7351 |  | A’ |
| 712.4 | 1.5232 | 0.2442 |  | A’’ |
| 733.2 | 7.8904 | 3.2796 |  | A’ |
| 734.95 | 0.0114 | 1.0077 |  | A’’ |
| 741.25 | 4.7485 | 4.4072 |  | A’ |
| 752.18 | 1.0646 | 31.0056 | Symmetric stretching (A_1_) in P-O_B_-P |  |
| 756.64 | 2.9368 | 3.1594 |  |  |
| 758.61 | 0.0069 | 4.7672 |  | A’’ |
| 773.16 | 4.5847 | 0.3997 |  |  |
| 773.4 | 1.8118 | 0.0403 |  | A’ |
| 776.46 | 1.7644 | 0.3608 |  | A’’ |
| 922.11 | 6.3712 | 76.5053 | Asymmetric stretching (B_1_) of 2(P-O_B_) in Q^2^ and asymmetric stretching (B_1_) in P-O_B_-P  (Q^2^-chains in [AlO_6_] environment) | A’ |
| 934.33 | 7.2226 | 172.2173 |  |  |
| 935.04 | 0.5563 | 7.5821 |  | A’’ |
| 978.02 | 9.3595 | 14.5303 |  |  |
| 993.85 | 2.0049 | 78.4574 |  |  |
| 998.52 | 5.1473 | 9.7822 |  | A’ |
| 1006.69 | 3.3938 | 7.7741 |  | A’’ |
| 1018.84 | 3.4457 | 3.6862 |  | A’ |
| 1020.94 | 2.771 | 274.8516 |  | A’’ |
| 1047.21 | 61.0491 | 2.4646 | Symmetric stretching (A_1_) of 2(P-O_NB_) and symmetric stretching (A_1_) of 2(P-O_B_) in Q^2^  (Q^2^-chains in [AlO_6_] environment) | A’ |
| 1070.02 | 6.6468 | 288.6196 |  |  |
| 1080.56 | 3.1604 | 0.3528 |  | A’’ |
| 1081.15 | 0.351 | 2.0535 |  | A’ |
| 1092.42 | 0.553 | 97.1632 |  | A’’ |
| 1095.28 | 0.0687 | 10.1482 |  |  |
| 1103.39 | 8.588 | 166.9846 |  | A’ |
| 1110.03 | 14.3523 | 4.0323 |  |  |
| 1113.09 | 4.5527 | 11.7003 |  | A’’ |
| 1116.74 | 38.7057 | 1.813 |  | A’ |
| 1118.15 | 1.2798 | 2.5219 |  | A’’ |
| 1126.53 | 15.8327 | 4.4306 |  | A’ |
| 1133.11 | 1.6103 | 0.1892 |  | A’’ |
| 1138.94 | 24.9022 | 3.4153 |  | A’ |
| 1141.84 | 4.8451 | 34.5021 |  | A’’ |
| 1145.41 | 11.1463 | 5.0046 |  | A’ |
| 1148.26 | 3.9255 | 0.5509 |  |  |
| 1148.93 | 12.9718 | 2.3152 |  | A’’ |
| 1159.02 | 2.5508 | 0.3928 | Symmetric stretching (A_1_) of 2(P-O_NB_) in Q^2^  (Q^2^-chains in [AlO_6_] environment) |  |
| 1163.71 | 5.9495 | 7.0589 |  | A’ |
| 1165.96 | 43.7341 | 0.0283 |  | A’’ |
| 1172.59 | 1.2926 | 5.4025 |  |  |
| 1173.06 | 202.9886 | 5.7915 |  | A’ |
| 1174.53 | 25.0037 | 0.3199 |  | A’’ |
| 1181.9 | 165.6491 | 3.726 |  | A’ |
| 1183.15 | 3.6085 | 2.1594 |  | A’’ |
| 1186.17 | 1369.7495 | 0.3144 |  |  |
| 1200.68 | 148.487 | 0.4427 | Asymmetric stretching (B_1_) of 2(P-O_NB_) in Q^2^ (Q^2^-chains in [AlO_6_] environment) | A’ |
| 1205.32 | 2.1491 | 0.4296 |  | A’’ |
| 1205.46 | 123.0199 | 0.6764 |  |  |
| 1207.26 | 41.2113 | 2.4425 |  | A’ |
| 1219.26 | 1.4762 | 223.5515 |  |  |
| 1221.96 | 1.4371 | 10.823 |  | A’’ |
| 1227.49 | 71.3288 | 1.5201 |  | A’ |
| 1230.72 | 3.804 | 1.092 |  | A’’ |
| 1235.65 | 3.9058 | 135.7389 |  | A’ |
| 1241.17 | 0.5545 | 89.9034 |  | A’’ |
| 1248.61 | 2.3224 | 91.2466 |  | A’ |
| 1250.32 | 8.776 | 95.3579 |  |  |
| 1252.54 | 0.4841 | 0.2782 |  | A’’ |
| 1311.05 | 0.9596 | 1.2016 |  |  |
| 1312.52 | 1.6748 | 0.4919 |  | A’ |
| 1322.51 | 3.4392 | 2.2589 |  |  |
| 1323.82 | 6.9301 | 0.4085 |  | A’’ |
| 1349.91 | 8.8385 | 0.0575 |  |  |

Non – nonactive, The most intense

Idealized Q^2^ unit have C_2v_ symmetry and $\Gamma_{osc}=4A_{1}+1A_{2}+2B_{1}+2B_{2}$ without rotations and translations. Fig. S1.3 shows vibrations of Q^2^ in B-Al(PO_3_)_3_ assigned to idealized Q^2^.

_
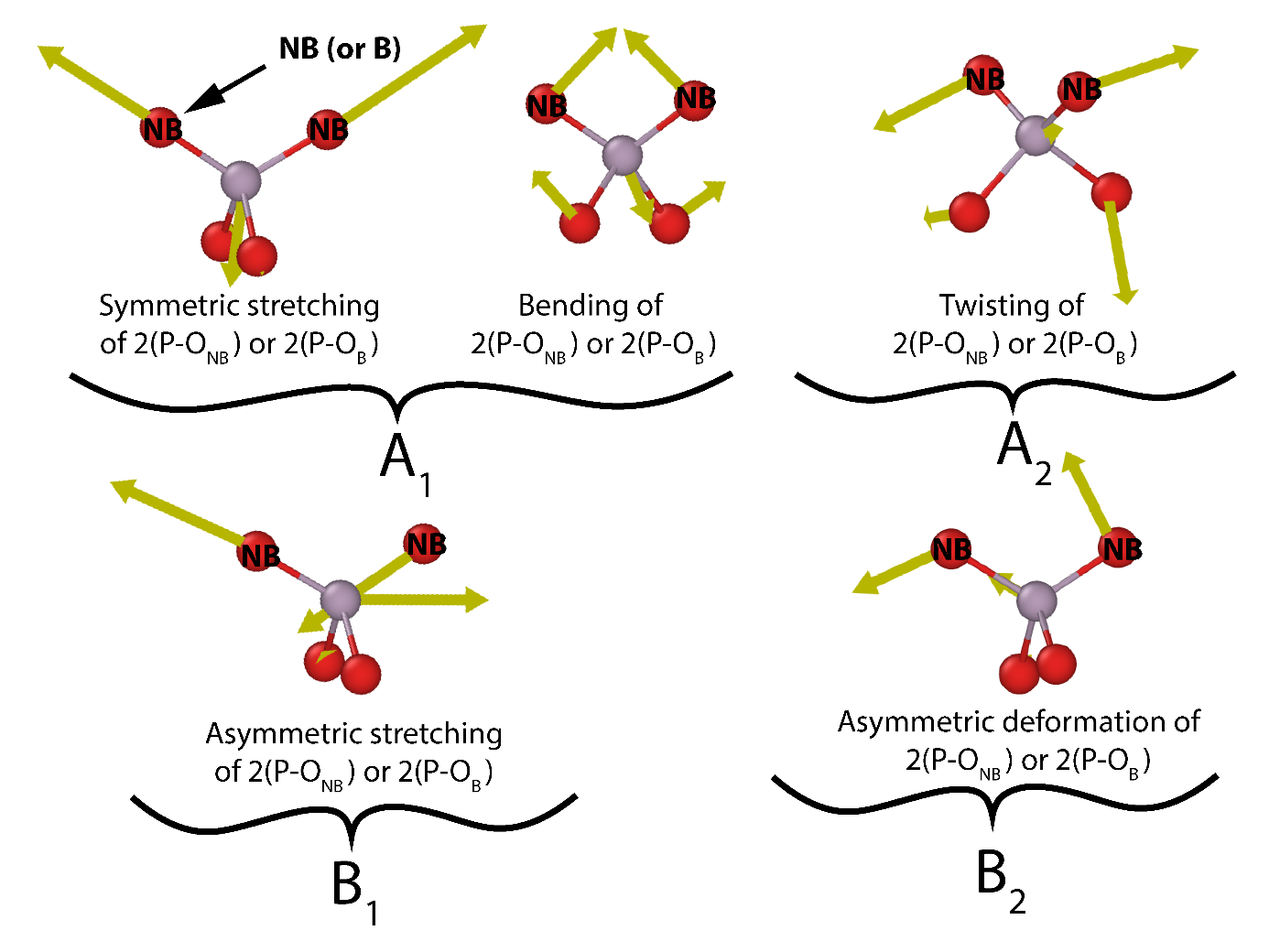
_

Fig. S1.3. Examples of vibrations in **B-Al(PO_3_)_3_** assignment to vibrations of idealized Q^2^ unit.

Two vibrations characteristic of the Raman spectrum for 4Q^2^-rings have been specified. 4Q^2^ ring point group is S_4_. The two vibrations symmetric and asymmetric about fourfold inversion axis was show in Fig. S1.4


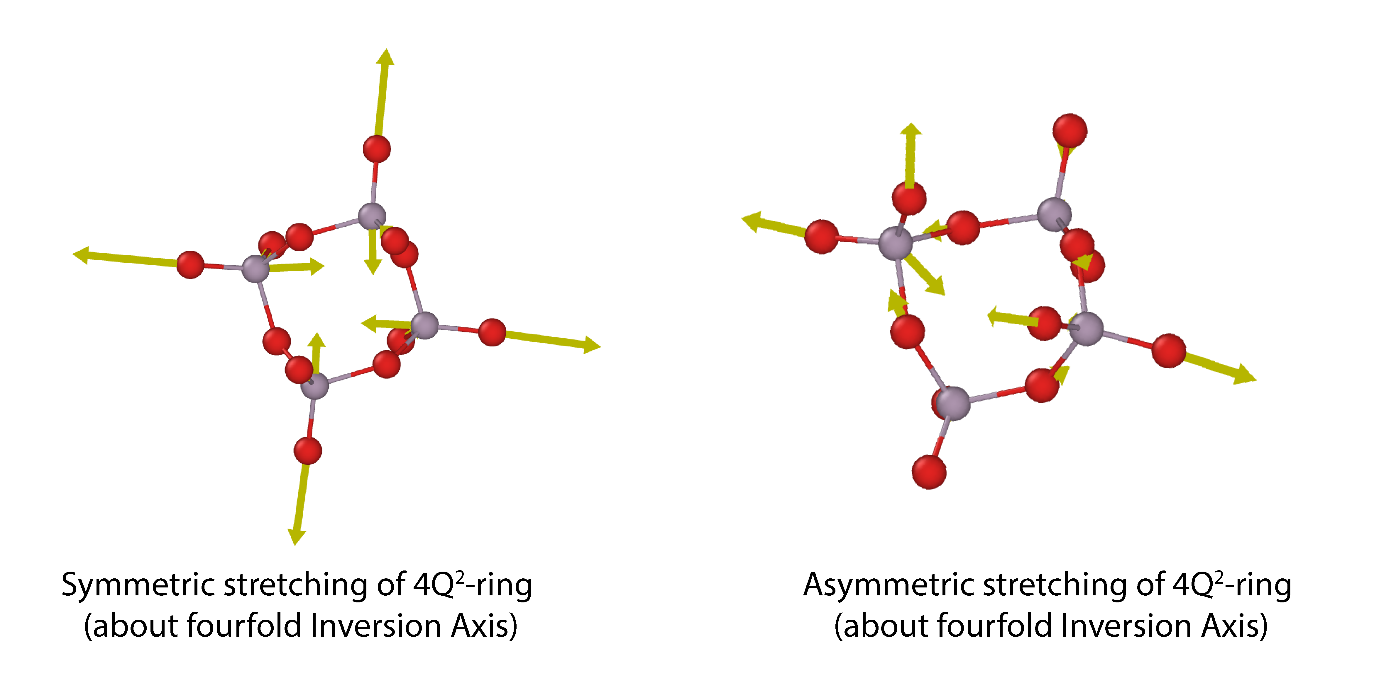


Fig. S1.4. Examples of vibrations of 4Q^2^-ring in **A-Al(PO_3_)_3_**.

**Table S1.3.** Calculated Raman and IR vibrations of **A-Al(PO_3_)_3_** (vibrations < 0.01 and nonactive in Raman and IR have been omitted).

| Frequency (>200) [cm^-1^] | Intensity Raman | Intensity IR | Assignment to Q^i^ idealized vibrations and P-O_B_-P | Vibrations in crystal (point group calculated as C_3_ (in real T_d_)) |
| --- | --- | --- | --- | --- |
| 201.13 | 1.4217 | 0.988 | Lattice vibrations and Librations | E |
| 204.51 | 2.4082 | 0.7433 |  | A |
| 204.6 | 0.9432 | 0.0035 |  | E |
| 206.95 | 0.0536 | 0.1214 |  | A |
| 213.64 | 0.8391 | 0.0524 |  | A |
| 213.69 | 3.0945 | 0.0054 |  | E |
| 217.05 | 2.1737 | 0.012 |  | E |
| 217.64 | 2.0923 | 0.0238 |  | A |
| 220.26 | 0.2555 | 0.0688 |  | A |
| 221.09 | 1.8988 | 1.2732 |  | E |
| 221.95 | 0.8388 | 0.0064 |  | A |
| 224.29 | 1.2024 | 0.098 |  | A |
| 224.32 | 0.2862 | 0 |  | E |
| 228.01 | 0.6802 | 0.2504 |  | A |
| 232.2 | 2.8628 | 0.0317 |  | A |
| 232.26 | 2.6951 | 0.2986 |  | E |
| 235.55 | 12.2669 | 0.1048 |  | E |
| 239.51 | 23.2883 | 0.0261 |  | A |
| 240.12 | 1.4518 | 0.0066 |  | E |
| 243.23 | 0.4462 | 0.0788 |  | E |
| 245.28 | 0.2411 | 5.9172 |  | A |
| 245.51 | 0.0935 | 2.3918 |  | A |
| 250.97 | 0.201 | 0.0916 |  | A |
| 251.55 | 2.2875 | 0.4948 |  | E |
| 256.19 | 2.1692 | 0.7031 |  | E |
| 258.05 | 0.4181 | 0.1616 |  | A |
| 259.52 | 3.8084 | 0.8971 |  | E |
| 262.07 | 0.0835 | 0.2048 |  | A |
| 263.11 | 2.7015 | 3.8296 |  | E |
| 267.09 | 2.0624 | 1.8049 |  | E |
| 268.34 | 0.9066 | 1.4448 |  | E |
| 269.43 | 0.235 | 2.2202 |  | A |
| 273.89 | 1.3668 | 0.0408 |  | A |
| 274.29 | 1.317 | 2.8676 |  | A |
| 274.96 | 2.2232 | 0.6486 |  | E |
| 277.2 | 15.0444 | 0.0594 |  | E |
| 280.32 | 0.5069 | 0.2609 |  | A |
| 281.85 | 0.2655 | 0.0146 |  | A |
| 283.03 | 3.4665 | 0.2226 |  | E |
| 285.73 | 19.5696 | 0.6301 | Deformations of 2(P-O_NB_) and 2(P-O_B_) (bending (A_1_) and asymmetric (B_2_)) in Q^2^ (4Q^2^-rings in [AlO_6_] environment) | E |
| 286.74 | 0.2485 | 0.0565 |  | A |
| 287.44 | 12.1964 | 0.4371 |  | E |
| 290.16 | 9.3725 | 0.3129 |  | A |
| 293.26 | 5.1161 | 0.1088 |  | A |
| 294.4 | 7.3271 | 0.4116 |  | A |
| 294.92 | 5.5097 | 0.1414 |  | E |
| 298.12 | 0.5306 | 0 |  | A |
| 298.54 | 4.7786 | 0.0651 |  | E |
| 302.53 | 6.0053 | 0.4042 |  | E |
| 305.87 | 2.3005 | 1.2566 |  | E |
| 306.16 | 5.5943 | 5.2503 |  | A |
| 306.94 | 0.6856 | 1.5101 |  | E |
| 310.9 | 0.3053 | 0.6292 |  | E |
| 312.76 | 0.644 | 0.3323 |  | A |
| 314.14 | 2.6847 | 0.0617 |  | E |
| 320.48 | 2.8824 | 0.1489 |  | A |
| 320.75 | 3.4097 | 0.4762 |  | E |
| 321.86 | 0.8182 | 0.0648 |  | A |
| 322.9 | 2.6218 | 0.5563 |  | E |
| 323.8 | 0.5925 | 0.0467 |  | E |
| 326.55 | 0.4318 | 0.1031 |  | E |
| 327.8 | 0.1351 | 0.0007 |  | A |
| 327.93 | 7.9129 | 0.0895 |  | E |
| 330.76 | 0.5093 | 0.4323 |  | E |
| 331.09 | 0.663 | 0.0792 |  | A |
| 334.84 | 0.9522 | 0.4831 |  | A |
| 336.1 | 0.1035 | 0.307 |  | E |
| 336.95 | 0.6396 | 0.2224 |  | A |
| 339.46 | 0.2135 | 0.0009 |  | A |
| 340.07 | 0.8568 | 1.0663 |  | E |
| 341.5 | 14.0034 | 0.0016 |  | A |
| 343.33 | 0.5223 | 0.1725 |  | E |
| 345.24 | 0.494 | 0.806 |  | A |
| 346.34 | 0.1548 | 4.0528 |  | E |
| 348.94 | 1.7052 | 21.3235 |  | E |
| 352.85 | 0.2282 | 15.2077 |  | E |
| 354.22 | 0.172 | 0.5819 |  | E |
| 357.87 | 0.007 | 2.2085 |  | E |
| 359.7 | 1.6661 | 0.8439 |  | A |
| 360.64 | 0.1277 | 12.9053 |  | E |
| 361.54 | 0.3042 | 5.4131 |  | E |
| 362.99 | 0.6189 | 0.1871 |  | A |
| 364.24 | 0.7254 | 0.4219 |  | A |
| 365.46 | 0.774 | 22.5814 | Bending (A_1_) of 2(P-O_B_) and 2(P-O_NB_) in Q^2^ (4Q^2^-rings in [AlO_6_] environment) | E |
| 368.2 | 0.367 | 11.6152 |  | E |
| 370.12 | 0.7565 | 45.9887 |  | E |
| 373.35 | 0.5075 | 25.0435 |  | E |
| 373.48 | 11.2746 | 1.5877 |  | A |
| 375.81 | 29.3556 | 0.0326 |  | A |
| 376.09 | 0.2857 | 6.9039 | Deformations of 2(P-O_NB_) and 2(P-O_B_) (bending (A_1_) and asymmetric (B_2_)) in Q^2^ (4Q^2^-rings in [AlO_6_] environment) | E |
| 377.02 | 1.056 | 0.0392 |  | A |
| 378.68 | 4.3112 | 0.0575 |  | A |
| 380.48 | 0.2678 | 13.6779 |  | E |
| 381.71 | 0.3307 | 2.1571 |  | A |
| 384.76 | 1.5417 | 25.0706 |  | E |
| 387.25 | 2.3104 | 24.973 |  | A |
| 388.74 | 0.7824 | 63.0193 |  | E |
| 389.41 | 0.9756 | 4.1843 |  | A |
| 390.96 | 0.7055 | 0.0311 |  | A |
| 391.9 | 5.9769 | 96.1229 |  | A |
| 393.55 | 1.8864 | 6.998 |  | E |
| 395.87 | 4.0851 | 0.8462 |  | E |
| 400.49 | 9.0976 | 1.1196 |  | E |
| 405.26 | 5.8557 | 2.3535 |  | E |
| 405.49 | 1.4224 | 2.6647 |  | A |
| 408.78 | 1.356 | 0.5821 |  | A |
| 409.71 | 13.3455 | 13.0885 |  | E |
| 412.15 | 3.8019 | 1.6912 |  | A |
| 414.55 | 0.2811 | 0.2907 |  | E |
| 415.06 | 1.4448 | 1.8407 | Asymmetric deformations (B_2_) of 2(P-O_NB_) and 2(P-O_B_) in Q^2^ and bending (A_1_) in P-O_B_-P (4Q^2^-rings in [AlO_6_] environment) | A |
| 416.09 | 1.7222 | 0.5819 |  | A |
| 418.55 | 8.4259 | 26.5362 |  | A |
| 419.96 | 0.8497 | 0.8001 |  | E |
| 420.94 | 3.441 | 3.8654 |  | A |
| 422.06 | 4.6579 | 0.6872 |  | E |
| 422.98 | 6.7223 | 12.7761 |  | A |
| 424.54 | 0.3241 | 27.9886 |  | A |
| 427.56 | 3.7613 | 8.1077 |  | E |
| 428.85 | 0.7514 | 57.6935 | Bending (A_1_) in P-O_B_-P and 2(P-O_NB_) in Q^2^ (4Q^2^-rings in [AlO_6_] environment) | A |
| 431.39 | 3.1188 | 62.1391 |  | A |
| 433.01 | 36.3825 | 4.4846 |  | A |
| 438.47 | 0.0853 | 0.4166 |  | E |
| 438.97 | 2.602 | 1.2967 |  | A |
| 439.72 | 0.1602 | 0.9513 |  | E |
| 440.37 | 1.353 | 2.0487 |  | A |
| 442.77 | 0.2136 | 0.0505 |  | E |
| 450.77 | 0.4935 | 0.076 |  | E |
| 453.04 | 0.7355 | 1.027 |  | A |
| 454.51 | 21.0737 | 5.4682 |  | A |
| 457.27 | 11.2617 | 4.9042 | Asymmetric deformations (B_2_) of 2(P-O_NB_) in Q^2^ and bending (A_1_) in P-O_B_-P (4Q^2^-rings in [AlO_6_] environment) | A |
| 460.98 | 8.338 | 0.0002 |  | A |
| 461.6 | 6.0746 | 2.1576 |  | A |
| 470.52 | 2.8831 | 1.9032 |  | E |
| 476.01 | 0.1755 | 0.2499 |  | A |
| 476.01 | 178.6098 | 0.2523 |  | E |
| 479.89 | 0.2014 | 0.0629 |  | E |
| 491.3 | 0.2951 | 0.1556 |  | A |
| 492.19 | 2.8401 | 0.0068 |  | A |
| 493.83 | 0.8896 | 0.0362 |  | A |
| 496.05 | 0.0342 | 0.1313 |  | E |
| 500.44 | 0.7544 | 0.0351 |  | A |
| 503.16 | 0.0639 | 0.1294 |  | E |
| 504.41 | 2.8233 | 0.9295 |  | A |
| 515.16 | 3.5382 | 0.036 |  | A |
| 519.11 | 0.4982 | 0.0942 |  | A |
| 522.83 | 3.4325 | 0.0002 |  | A |
| 526.72 | 0.3748 | 0.0002 |  | A |
| 526.99 | 5.4716 | 0.2288 |  | E |
| 530.3 | 0.1089 | 0.3263 |  | E |
| 534.55 | 0.1633 | 0.8276 |  | E |
| 536.25 | 0.0422 | 0.1186 |  | A |
| 541.21 | 0.348 | 3.4281 |  | E |
| 546.69 | 0.9318 | 0.2624 |  | E |
| 555.73 | 0.0918 | 2.3533 |  | E |
| 560.41 | 0.1631 | 4.3182 |  | E |
| 561.54 | 0.0089 | 0.2664 |  | E |
| 562.96 | 3.9891 | 0.1075 |  | A |
| 565.32 | 0.5992 | 8.2958 |  | E |
| 565.99 | 0.3291 | 0.4971 |  | A |
| 567.27 | 0.407 | 3.8397 |  | E |
| 569.72 | 0.6656 | 9.7939 |  | E |
| 570.77 | 0.5933 | 0.0318 |  | A |
| 571.41 | 0.3266 | 0.3056 |  | A |
| 575.76 | 0.3657 | 1.6413 |  | A |
| 576.69 | 0.4787 | 0.0004 |  | A |
| 580.57 | 0.6248 | 10.6407 |  | E |
| 584.32 | 0.1118 | 1.4677 |  | E |
| 592.99 | 0.0513 | 1.2482 |  | E |
| 594.5 | 0.016 | 0.3889 |  | E |
| 595.38 | 0.9504 | 0.0245 |  | A |
| 601.22 | 12.431 | 6.2689 |  | A |
| 605.2 | 60.0496 | 0.1368 |  | A |
| 607.75 | 1.0139 | 0.5628 | Asymmetric deformations (B_2_) of 2(P-O_NB_) and 2(P-O_B_) in Q^2^ (4Q^2^-rings in [AlO_6_] environment) | A |
| 612.07 | 84.9479 | 0.4748 |  | A |
| 618.93 | 19.9006 | 0.0043 |  | A |
| 621.92 | 11.568 | 8.6391 |  | A |
| 628 | 4.6688 | 0.0222 |  | A |
| 630.39 | 11.707 | 0.032 |  | A |
| 650.5 | 146.4604 | 0.0809 |  | A |
| 654.18 | 361.1509 | 0.0595 | Symmetric stretching (A_1_) in P-O_B_-P and bending (A_1_) of 2(P-O_NB_) in Q^2^  (4Q^2^-rings in [AlO_6_] environment) | A |
| 659.17 | 290.8995 | 0.1004 |  | A |
| 667.99 | 1.5955 | 0.0424 |  | A |
| 670.44 | 7.7728 | 0.1262 |  | A |
| 672.4 | 0.0549 | 0.1087 |  | E |
| 677.21 | 0.0053 | 0.1306 |  | E |
| 678.53 | 0.0381 | 0.3544 |  | E |
| 704.73 | 0.0275 | 0.4201 |  | E |
| 707.86 | 0.08 | 3.5017 | Symmetric stretching (B_1_) in P-O_B_-P (4Q^2^-rings in [AlO_6_] environment) | E |
| 710.08 | 0.2751 | 18.4646 |  | E |
| 712.56 | 0.2855 | 18.0687 |  | E |
| 713.93 | 1.4632 | 18.3951 |  | A |
| 719.53 | 0.1227 | 15.3474 |  | E |
| 727.2 | 0.2266 | 0.8027 |  | A |
| 729.47 | 0.8775 | 19.7185 |  | A |
| 730.12 | 0.0032 | 0.1054 |  | E |
| 732.54 | 0.2043 | 1.4575 |  | A |
| 734.3 | 1.6826 | 2.45 |  | A |
| 735.88 | 0.8721 | 10.8699 |  | A |
| 737.45 | 0.2546 | 3.162 |  | A |
| 740.7 | 1.0811 | 0.638 |  | A |
| 742.78 | 0.0269 | 0.3884 |  | E |
| 767.65 | 0.4717 | 3.8523 |  | E |
| 793.59 | 0.1696 | 6.8616 |  | A |
| 799.17 | 0.0806 | 0.7951 |  | A |
| 799.32 | 9.4904 | 0.0544 |  | E |
| 801.13 | 0.0266 | 0.3027 |  | A |
| 886.18 | 8.8626 | 0.0201 | Asymmetric stretching (B_1_) in P-O_B_-P (4Q^2^-rings in [AlO_6_] environment) | E |
| 894.47 | 10.5938 | 0.0552 |  | A |
| 894.91 | 6.5935 | 0.4094 |  | A |
| 895.85 | 2.3062 | 0.0033 |  | A |
| 897.1 | 1.4129 | 0.6215 |  | A |
| 985.55 | 9.7739 | 256.6429 |  | A |
| 986.96 | 10.6807 | 251.631 | Asymmetric stretching (B_1_) and symmetric stretching (A_1_) of 2(P-O_B_) in Q^2^ in different positions and asymmetric stretching (B_1_) in P-O_B_-P (4Q^2^-rings in [AlO_6_] environment) | E |
| 991.4 | 0.1895 | 2.8473 |  | E |
| 992.99 | 0.0821 | 0.6024 |  | E |
| 1001.14 | 0.0577 | 0.1457 |  | A |
| 1002.37 | 0.0453 | 0.3527 |  | E |
| 1006.4 | 0.3097 | 0.004 |  | A |
| 1010.2 | 0.4303 | 1.2182 |  | A |
| 1025.03 | 2.6874 | 163.8193 |  | E |
| 1029.83 | 0.1834 | 23.1678 |  | A |
| 1031.09 | 0.2923 | 15.0735 |  | A |
| 1032.71 | 0.8465 | 121.1365 |  | A |
| 1035.03 | 0.0593 | 1.9629 |  | E |
| 1035.34 | 0.2048 | 0.6357 |  | A |
| 1036.96 | 0.0049 | 0.0128 |  | E |
| 1046.33 | 0.0499 | 0.5051 |  | E |
| 1061.23 | 1.3069 | 28.6464 | Symmetric stretching (A_1_) of 2(P-O_B_) in Q^2^ (4Q^2^-rings in [AlO_6_] environment) | A |
| 1064.27 | 1.3582 | 31.5276 | Symmetric stretching (A_1_) of 2(P-O_NB_) and Symmetric stretching (A_1_) of 2(P-O_B_) in Q^2^ (4Q^2^-rings in [AlO_6_] environment) | E |
| 1082.05 | 70.9237 | 0.0278 |  | E |
| 1090.56 | 0.007 | 0.1332 |  | A |
| 1091.64 | 0.0926 | 0.2324 |  | A |
| 1142.81 | 0.5227 | 0.1182 |  | A |
| 1149.37 | 6.3226 | 18.4674 |  | A |
| 1159.27 | 1.7459 | 0.8212 | Symmetric stretching (A_1_) of 2(P-O_NB_) in Q^2^ (4Q^2^-rings in [AlO_6_] environment) | A |
| 1173.26 | 5.7238 | 20.788 |  | A |
| 1174.01 | 1.5571 | 5.161 |  | A |
| 1176.4 | 30.2347 | 0.107 |  | E |
| 1181.78 | 4.5249 | 0.1422 |  | A |
| 1181.87 | 8.8642 | 0.0201 |  | A |
| 1183.24 | 4.8721 | 0.0004 |  | A |
| 1183.44 | 0.3706 | 0.5649 |  | E |
| 1184.52 | 14.022 | 0.0281 |  | A |
| 1186.32 | 4.2271 | 0.4771 |  | A |
| 1187.58 | 6.1225 | 0.0036 |  | A |
| 1187.59 | 0.724 | 5.1445 |  | E |
| 1196.52 | 0.4234 | 27.9911 |  | E |
| 1198.86 | 0.1504 | 2.1434 |  | E |
| 1200.13 | 0.1731 | 1.4848 |  | E |
| 1215.1 | 12.0194 | 0.0097 |  | A |
| 1217.44 | 0.3728 | 0.007 |  | A |
| 1218.55 | 7.2064 | 7.9102 |  | E |
| 1220.26 | 90.1033 | 0.0846 |  | A |
| 1223.36 | 0.1178 | 0.4251 |  | E |
| 1225.51 | 819.8139 | 0.0518 | Symmetric stretching (A_1_) of 2(P-O_NB_) in Q^2^ (4Q^2^-rings in [AlO_6_] environment) and Stretching asymmetrical about fourfold inversion axis of the 4Q^2^ ring | A |
| 1225.83 | 23.7582 | 3.7438 | Symmetric stretching (A_1_) of 2(P-O_NB_) in Q^2^ (4Q^2^-rings in [AlO_6_] environment) | E |
| 1226.78 | 4.262 | 0.7631 |  | E |
| 1234.48 | 52.4054 | 0.0092 |  | E |
| 1234.95 | 1212.831 | 0.2332 |  | A |
| 1240.12 | 7.2174 | 0.4499 |  | E |
| 1252.15 | 0.2354 | 0.3018 | Asymmetric stretching (B_1_) of 2(P-O_NB_) in Q^2^ (4Q^2^-rings in [AlO_6_] environment) | E |
| 1266.37 | 9.0693 | 107.2843 |  | A |
| 1266.73 | 1.3217 | 0.1017 |  | E |
| 1267.9 | 1.8187 | 0.2935 |  | E |
| 1269.67 | 60.025 | 0.8889 | Stretching asymmetrical to the center of the 4Q^2^ ring | E |
| 1269.75 | 0.2209 | 0.0497 | Asymmetric stretching (B_1_) of 2(P-O_NB_) in Q^2^ (4Q^2^-rings in [AlO_6_] environment) | A |
| 1269.88 | 0.1205 | 0.0306 |  | A |
| 1270.44 | 0.1473 | 0.2091 |  | A |
| 1273.4 | 1.0132 | 0.712 |  | E |
| 1274.03 | 5.8787 | 62.9964 |  | E |
| 1274.52 | 2.5137 | 21.2454 |  | E |
| 1277.07 | 0.4924 | 0.0807 |  | E |
| 1279.89 | 0.1387 | 0.0841 |  | E |
| 1280.71 | 0.2569 | 0.2229 |  | E |
| 1282.79 | 55.3206 | 1.5297 | Stretching symmetrical to the center of the 4Q^2^ ring | E |
| 1285.16 | 0.2432 | 5.5147 | Asymmetric stretching (B_1_) of 2(P-O_NB_) in Q^2^ (4Q^2^-rings in [AlO_6_] environment) | A |
| 1285.61 | 2.5359 | 71.0901 |  | A |
| 1285.73 | 5.0702 | 141.2571 |  | A |
| 1286.85 | 0.0335 | 0.171 |  | A |
| 1289.25 | 9.4013 | 232.7527 |  | E |
| 1295 | 340.184 | 0.0671 | Asymmetric stretching (B_1_) of 2(P-O_NB_) in Q^2^ (4Q^2^-rings in [AlO_6_] environment) and stretching symmetrical about fourfold inversion axis of the 4Q^2^ ring | A |
| 1296.77 | 0.3384 | 1.6421 | Asymmetric stretching (B_1_) of 2(P-O_NB_) in Q^2^ (4Q^2^-rings in [AlO_6_] environment) | E |
| 1301.25 | 3.605 | 0.0134 |  | A |
| 1307.63 | 0.1224 | 0.2409 |  | E |
| 1308.75 | 0.0176 | 0.2312 |  | E |
| 1322.65 | 7.8793 | 0.7106 |  | E |
| 1386.54 | 0.3354 | 0 |  | A |
| 1405.37 | 11.31 | 0.0003 |  | A |

Non – nonactive, The most intense

Some vibrations characteristic of the Raman spectrum for 6Q^2^-rings have been specified in figure S1.5. 6Q^2^-ring have S_i_ point group and vibrations symmetric about center of inversion A_g_ and asymmetric A_u_.


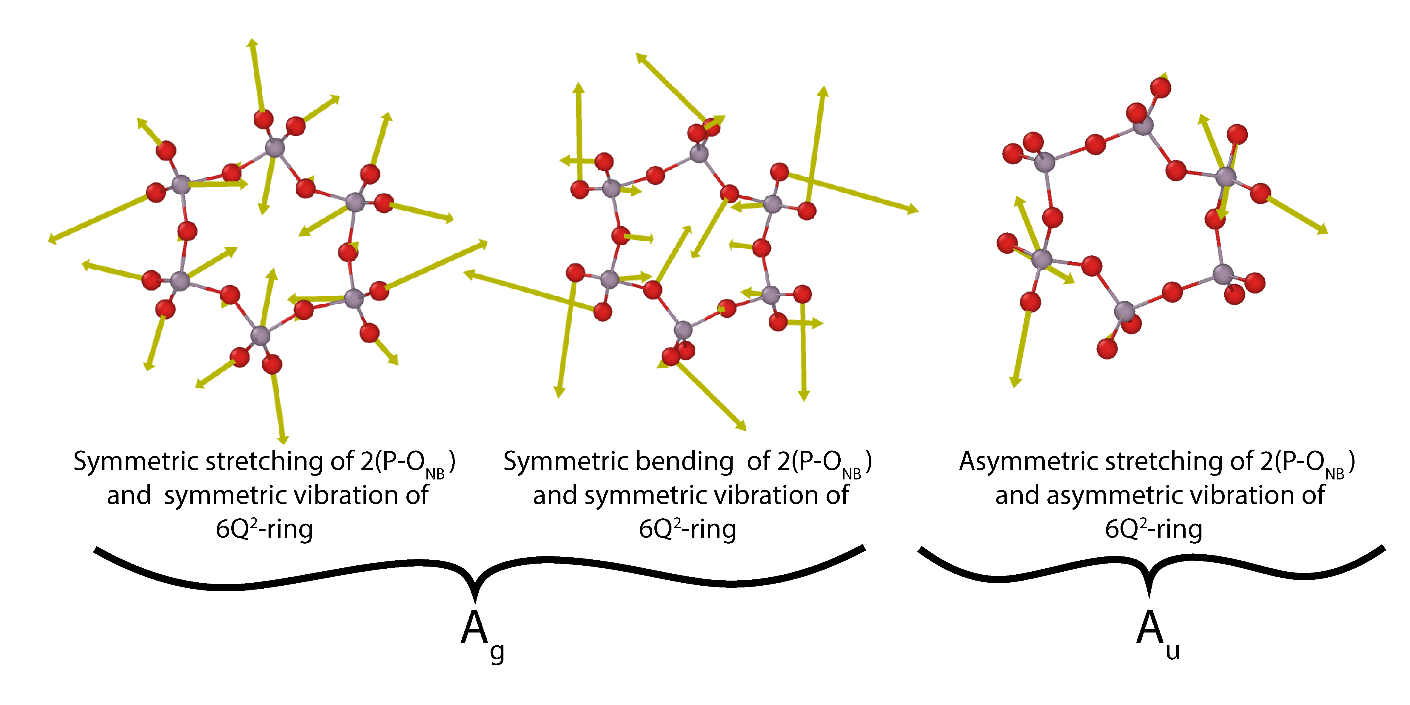


Fig. S1.5. Examples of vibrations of 6Q^2^-ring in **Aluminum cyclohexaphosphate -Al(PO_3_)_3_**.

**Table S1.4.** Calculated Raman and IR vibrations of **aluminum cyclohexaphosphate -Al(PO_3_)_3_** (vibrations < 0.01 and nonactive in Raman and IR have been omitted).

| Frequency (>200) [cm^-1^] | Intensity Raman | Intensity IR | Assignment to Q^i^ idealized vibrations and P-O_B_-P | Vibrations in crystal (point group C_2h_) |
| --- | --- | --- | --- | --- |
| 214.41 | Non | 0.2467 | Lattice vibrations and librations | B_u_ |
| 227.81 | 1.4663 | Non |  | B_g_ |
| 228.64 | Non | 0.0082 |  | A_u_ |
| 234.1 | 1.6371 | Non |  | A_g_ |
| 240.31 | Non | 0.3014 |  | B_u_ |
| 249.34 | 3.5007 | Non |  | B_g_ |
| 253.91 | 9.0889 | Non |  | A_g_ |
| 254.52 | Non | 1.7928 |  | B_u_ |
| 263.78 | Non | 1.7567 |  | A_u_ |
| 265.28 | 2.2328 | Non | Asymmetric deformations (B_2_) of 2(P-O_NB_) and 2(P-O_B_) in Q^2^ (6Q^2^-rings in [AlO_6_] environment | B_g_ |
| 268.78 | Non | 0.4013 |  | B_u_ |
| 272.97 | Non | 0.3012 |  | A_u_ |
| 273.3 | Non | 1.9273 |  | B_u_ |
| 276.1 | 6.9518 | Non |  | A_g_ |
| 276.71 | 1.1303 | Non |  | B_g_ |
| 287.12 | 11.9192 | Non |  | A_g_ |
| 288.12 | Non | 0.0664 |  | B_u_ |
| 290.44 | Non | 0.7274 |  | A_u_ |
| 294.89 | 0.195 | Non |  | B_g_ |
| 295.56 | Non | 0.0195 |  | A_u_ |
| 298.64 | 1.9246 | Non |  | A_g_ |
| 307.74 | 19.0387 | Non |  | A_g_ |
| 307.87 | Non | 0.0506 |  | B_u_ |
| 308.41 | 0.8636 | Non |  | B_g_ |
| 310.64 | Non | 0.0006 |  | A_u_ |
| 312.91 | Non | 0.3891 |  | A_u_ |
| 329.51 | 9.2693 | Non |  | A_g_ |
| 334.76 | Non | 5.0757 |  | B_g_ |
| 334.79 | 0.3739 | Non |  | B_u_ |
| 341.7 | Non | 3.3641 |  | B_u_ |
| 351.83 | 3.3894 | Non |  | B_g_ |
| 354.25 | Non | 0.24 |  | A_u_ |
| 355.32 | Non | 2.0004 |  | B_u_ |
| 363.95 | 3.3906 | Non |  | A_g_ |
| 366.94 | 8.4821 | Non |  | A_g_ |
| 367.94 | 0.9104 | Non |  | B_g_ |
| 369.16 | 3.5994 | Non |  | A_g_ |
| 370.1 | Non | 3.1017 | Asymmetric deformations (B_2_) of 2(P-O_NB_) and 2(P-O_B_) in Q^2^ and bending (A_1_) in P-O_B_-P (6Q^2^-rings in [AlO_6_] environment) | B_u_ |
| 373.06 | Non | 0.0365 | Asymmetric deformations (B_2_) of 2(P-O_NB_) and 2(P-O_B_) in Q^2^ (6Q^2^-rings in [AlO_6_] environment) | A_u_ |
| 376.59 | Non | 2.6971 |  | A_u_ |
| 387.29 | 6.0262 | Non |  | A_g_ |
| 388.6 | 3.749 | Non | Bending (A_1_) and asymmetric deformations (B_2_) of 2(P-O_NB_) and 2(P-O_B_) in Q^2^ (6Q^2^-rings in [AlO_6_] environment) | B_g_ |
| 389.27 | Non | 9.5525 |  | B_u_ |
| 389.38 | Non | 13.0968 |  | A_u_ |
| 393.6 | 1.6511 | Non |  | B_g_ |
| 402.41 | Non | 22.936 |  | B_u_ |
| 408.27 | 9.1976 | Non | Twisting (A_2_) of 2(P-O_NB_) and 2(P-O_B_) in Q^2^ (6Q^2^-rings in [AlO_6_] environment) | A_g_ |
| 418.37 | 6.2711 | Non |  | B_g_ |
| 420.46 | Non | 0.5482 | Asymmetric deformations (B_2_) of 2(P-O_NB_) and 2(P-O_B_) in Q^2^ (6Q^2^-rings in [AlO_6_] environment) | A_u_ |
| 420.87 | 0.7985 | Non |  | A_g_ |
| 423.43 | Non | 31.1204 |  | A_u_ |
| 423.45 | Non | 16.6959 |  | B_u_ |
| 447.93 | 10.9153 | Non |  | B_g_ |
| 449.63 | 6.0876 | Non |  | B_g_ |
| 460.2 | 5.2903 | Non |  | A_g_ |
| 474.2 | Non | 38.1954 | Bending (A_1_) and asymmetric deformations (B_2_) of 2(P-O_NB_) and 2(P-O_B_) in Q^2^ (6Q^2^-rings in [AlO_6_] environment) | B_u_ |
| 485.23 | 1.4672 | Non |  | A_g_ |
| 487.64 | Non | 19.6246 |  | A_u_ |
| 500.31 | Non | 20.4614 | Bending (A_1_) and asymmetric deformations (B_2_) of 2(P-O_NB_) in Q^2^ (6Q^2^-rings in [AlO_6_] environment) | A_u_ |
| 506.96 | Non | 5.0461 |  | B_u_ |
| 512.03 | 0.8545 | Non |  | B_g_ |
| 521.24 | Non | 1.1715 |  | A_u_ |
| 528.59 | Non | 15.4276 |  | B_u_ |
| 531.4 | 0.289 | Non |  | B_g_ |
| 532.27 | 2.621 | Non |  | A_g_ |
| 541.3 | 13.7439 | Non |  | A_g_ |
| 546.98 | Non | 28.8243 | Asymmetric deformation (B_2_) of 2(P-O_NB_) in Q^2^ (6Q^2^-rings in [AlO_6_] environment) | B_u_ |
| 547.74 | Non | 11.034 |  | A_u_ |
| 555.33 | 0.1495 | Non | Twisting (A_2_) of 2(P-O_NB_) and 2(P-O_B_) in Q^2^ (6Q^2^-rings in [AlO_6_] environment) | B_g_ |
| 558.81 | 97.5682 | Non | Twisting (A_2_) of 2(P-O_NB_) and 2(P-O_B_) in Q^2^ (6Q^2^-rings in [AlO_6_] environment) and Symmetric A_g_ vibration of 6Q^2^ molecule | A_g_ |
| 565.09 | 0.3813 | Non | Asymmetric deformation (B_2_) and bending (A_1_)of 2(P-O_NB_) in Q^2^ in different positions (6Q^2^-rings in [AlO_6_] environment) | B_g_ |
| 565.3 | Non | 4.37 |  | A_u_ |
| 566.71 | Non | 4.032 |  | B_u_ |
| 570.9 | Non | 12.1475 |  | B_u_ |
| 572.9 | Non | 9.5128 |  | A_u_ |
| 577.82 | 12.0886 | Non |  | A_g_ |
| 585.02 | 2.0288 | Non |  | B_g_ |
| 593.8 | Non | 1.9065 | Asymmetric deformation (B_2_) of 2(P-O_NB_) in Q^2^ (6Q^2^-rings in [AlO_6_] environment) | A_u_ |
| 596.79 | Non | 12.2139 | Bending (A_1_) in P-O_B_-P (6Q^2^-rings in [AlO_6_] environment) | B_u_ |
| 613.29 | 13.8976 | Non | Bending (A_1_) in P-O_B_-P and asymmetric deformation (B_2_) of 2(P-O_NB_) in Q^2^ (6Q^2^-rings in [AlO_6_] environment) | A_g_ |
| 625.52 | 2.6572 | Non | Bending (A_1_) in P-O_B_-P (6Q^2^-rings in [AlO_6_] environment) | B_g_ |
| 635.96 | 1.653 | Non |  | B_g_ |
| 644.78 | 13.1649 | Non |  | A_g_ |
| 647.05 | Non | 3.8959 |  | B_u_ |
| 656.17 | Non | 1.011 |  | A_u_ |
| 690.24 | Non | 0.3092 | Symmetric stretching (A_1_) in P-O_B_-P and bending (A_1_) of 2(P-O_NB_) in Q^2^ (6Q^2^-rings in [AlO_6_] environment) | A_u_ |
| 697.01 | Non | 0.1576 |  | B_u_ |
| 714.94 | 212.181 | Non |  | A_g_ |
| 718.52 | 2.4161 | Non |  | B_g_ |
| 725.2 | Non | 0.8643 |  | B_u_ |
| 733.21 | Non | 0.0107 |  | A_u_ |
| 747.55 | 47.1051 | Non |  | A_g_ |
| 749.79 | 1.3119 | Non |  | B_g_ |
| 770.12 | 3.3497 | Non | Symmetric stretching (A_1_) in P-O_B_-P (6Q^2^-rings in [AlO_6_] environment) | A_g_ |
| 771.75 | 2.7078 | Non |  | B_g_ |
| 790.09 | Non | 6.4973 |  | A_u_ |
| 790.52 | Non | 12.1123 |  | B_u_ |
| 882.74 | 0.4859 | Non | Asymmetric stretching (B_1_) in P-O_B_-P (6Q^2^-rings in [AlO_6_] environment) | A_g_ |
| 886.04 | 2.3134 | Non |  | B_g_ |
| 937.01 | Non | 56.3711 |  | A_u_ |
| 945.52 | Non | 140.1475 |  | B_u_ |
| 974.11 | Non | 46.4295 | Symmetric (A_1_) and asymmetric (B_1_) stretching of 2(P-O_B_) in different Q^2^ positions (6Q^2^-rings in [AlO_6_] environment) | A_u_ |
| 1004.75 | 12.8464 | Non | Symmetric (A_1_) stretching (A_1_) of 2(P-O_B_) in Q^2^ positions (6Q^2^-rings in [AlO_6_] environment) | A_g_ |
| 1015.23 | Non | 51.5631 | Asymmetric stretching (B_1_) in P-O_B_-P (6Q^2^-rings in [AlO_6_] environment) | B_u_ |
| 1015.3 | 2.8334 | Non | Symmetric (A_1_) stretching (A_1_) of 2(P-O_B_) in Q^2^ positions (6Q^2^-rings in [AlO_6_] environment) and Symmetric A_g_ vibration of 6Q^2^ molecule | B_g_ |
| 1068.08 | Non | 33.9706 | Symmetric stretching (A_1_) of 2(P-O_B_) in Q^2^ positions (6Q^2^-rings in [AlO_6_] environment) and Asymmetric A_u_ vibration of 6Q^2^ molecule | B_u_ |
| 1068.72 | Non | 10.0975 |  | A_u_ |
| 1071.33 | 15.7874 | Non | Symmetric (A_1_) and asymmetric (B_1_) stretching of 2(P-O_B_) in different Q^2^ positions (6Q^2^-rings in [AlO_6_] environment) | A_g_ |
| 1081.94 | 3.3013 | Non |  | B_g_ |
| 1087.74 | Non | 2.7823 | Symmetric stretching (A_1_) of 2(P-O_NB_) in Q^2^ (6Q^2^-rings in [AlO_6_] environment) | B_u_ |
| 1096.61 | 5.8992 | Non |  | A_g_ |
| 1101.02 | 4.959 | Non |  | B_g_ |
| 1102.16 | Non | 4.5576 |  | A_u_ |
| 1121.44 | 12.052 | Non | Symmetric stretching (A_1_) of 2(P-O_NB_) in Q^2^ (6Q^2^-rings in [AlO_6_] environment) and Symmetric A_g_ vibration of 6Q^2^ molecule | A_g_ |
| 1123.77 | 9.1159 | Non |  | B_g_ |
| 1146.15 | Non | 0.1453 | Symmetric stretching (A_1_) of 2(P-O_NB_) in Q^2^ (6Q^2^-rings in [AlO_6_] environment) and Asymmetric A_u_ vibration of 6Q^2^ molecule | A_u_ |
| 1148.68 | Non | 21.2383 |  | B_u_ |
| 1164.62 | Non | 2.4967 |  | A_u_ |
| 1183.1 | Non | 32.3709 |  | B_u_ |
| 1205.15 | 23.1273 | Non | Symmetric stretching (A_1_) of 2(P-O_NB_) in Q^2^ (6Q^2^-rings in [AlO_6_] environment) and Symmetric A_g_ vibration of 6Q^2^ molecule | B_g_ |
| 1214.51 | 457.2895 | Non |  | A_g_ |
| 1221.65 | 29.0114 | Non | Asymmetric stretching (B_1_) of 2(P-O_NB_) in Q^2^ (6Q^2^-rings in [AlO_6_] environment) and Symmetric A_g_ vibration of 6Q^2^ molecule | A_g_ |
| 1235.87 | 12.2616 | Non |  | B_g_ |
| 1258.72 | Non | 69.5105 | Asymmetric stretching (B_1_) of 2(P-O_NB_) in Q^2^ (6Q^2^-rings in [AlO_6_] environment) | A_u_ |
| 1264.82 | Non | 32.9708 |  | B_u_ |
| 1265.74 | Non | 71.7603 |  | B_u_ |
| 1266.64 | Non | 5.6132 | Asymmetric stretching (B_1_) of 2(P-O_NB_) in Q^2^ (6Q^2^-rings in [AlO_6_] environment) | A_u_ |
| 1267.97 | 10.644 | Non | Asymmetric stretching (B_1_) of 2(P-O_NB_) in Q^2^ (6Q^2^-rings in [AlO_6_] environment) and Symmetric A_g_ vibration of 6Q^2^ molecule | B_g_ |
| 1268.64 | 27.8453 | Non |  | A_g_ |
| 1281.83 | 110.5328 | Non |  | A_g_ |
| 1313.54 | Non | 16.2822 | Asymmetric stretching (B_1_) of 2(P-O_NB_) in Q^2^ (6Q^2^-rings in [AlO_6_] environment) | B_u_ |
| 1329.26 | Non | 4.848 |  | A_u_ |
| 1341.4 | 32.882 | Non | Asymmetric stretching (B_1_) of 2(P-O_NB_) in Q^2^ (6Q^2^-rings in [AlO_6_] environment) and Symmetric A_g_ vibration of 6Q^2^ molecule | B_g_ |

Non – nonactive, The most intense

**Table S1.5.** Calculated Raman and IR vibrations of **NaAlP_2_O_7_**. (vibrations < 0.01 and nonactive in Raman and IR have been omitted).

| Frequency (>200) [cm^-1^] | Intensity Raman | Intensity IR | Assignment to Q^i^ idealized vibrations and P-O_B_-P | Vibrations in crystal (point group C_2h_) |
| --- | --- | --- | --- | --- |
| 207.55 | 0.1064 | Non | Lattice vibrations and librations | B_g_ |
| 221.44 | 0.5335 | Non |  | B_g_ |
| 223.31 | Non | 0.958 |  | B_u_ |
| 227.12 | Non | 0.3031 |  | A_u_ |
| 239.2 | Non | 0.8101 |  | B_u_ |
| 240.02 | 4.593 | Non |  | A_g_ |
| 243.42 | Non | 0.0853 |  | A_u_ |
| 247.05 | Non | 0.1807 |  | B_u_ |
| 263.37 | Non | 1.1434 |  | A_u_ |
| 263.92 | 0.0881 | Non |  | B_g_ |
| 265.64 | 1.9287 | Non |  | A_g_ |
| 276.68 | Non | 0.1166 |  | A_u_ |
| 282.71 | 1.1065 | 0 |  | B_g_ |
| 291.72 | Non | 2.842 |  | B_u_ |
| 292.14 | Non | 1.7459 |  | A_u_ |
| 296.19 | 7.2735 | Non |  | A_g_ |
| 313.81 | 1.428 | Non |  | B_g_ |
| 320.09 | Non | 7.4165 |  | A_u_ |
| 329.24 | Non | 2.606 |  | B_u_ |
| 333.01 | 0.5123 | Non |  | B_g_ |
| 340.85 | 10.8508 | Non | Asymmetric (E) deformations of 3(P-O_NB_) (in [AlO_6_] and Na^+^ environment) | A_g_ |
| 346.12 | Non | 0.2625 |  | A_u_ |
| 352.83 | 0.3012 | Non |  | B_g_ |
| 353.36 | Non | 4.3106 |  | A_u_ |
| 353.8 | Non | 12.1373 |  | B_u_ |
| 357.61 | 6.7157 | Non |  | A_g_ |
| 368.06 | 5.3833 | Non |  | A_g_ |
| 378.95 | Non | 20.2458 |  | B_u_ |
| 384.83 | Non | 55.9059 |  | B_u_ |
| 386.93 | 7.691 | Non |  | A_g_ |
| 395.97 | Non | 0.014 |  | A_u_ |
| 396.46 | 0.4197 | Non |  | B_g_ |
| 404.19 | Non | 27.2471 |  | A_u_ |
| 409.09 | 3.0536 | Non |  | B_g_ |
| 413.47 | 5.4933 | Non |  | A_g_ |
| 424.13 | Non | 13.6106 |  | B_u_ |
| 433.36 | 6.047 | Non |  | A_g_ |
| 441.9 | Non | 21.969 |  | A_u_ |
| 444.38 | 1.8696 | Non |  | B_g_ |
| 475.67 | 4.0541 | Non |  | B_g_ |
| 478.26 | Non | 0.0467 |  | B_u_ |
| 481.98 | Non | 1.3749 |  | B_u_ |
| 489.52 | Non | 0.9088 |  | A_u_ |
| 496.49 | 6.3691 | Non |  | B_g_ |
| 499.41 | Non | 37.827 |  | B_u_ |
| 504.04 | 29.5987 | Non |  | A_g_ |
| 512.28 | 2.1659 | Non |  | B_g_ |
| 517.6 | 19.778 | Non |  | A_g_ |
| 521.25 | Non | 0.0141 |  | A_u_ |
| 534.59 | 3.892 | Non |  | B_g_ |
| 546.88 | Non | 2.1593 |  | A_u_ |
| 549.43 | 8.5052 | Non |  | A_g_ |
| 552.43 | Non | 10.8018 |  | B_u_ |
| 564.26 | Non | 0.5699 |  | B_u_ |
| 567.14 | 14.0344 | Non |  | A_g_ |
| 568.63 | Non | 1.6073 |  | A_u_ |
| 573.93 | 0.1641 | Non |  | B_g_ |
| 574.15 | 22.9514 | Non |  | A_g_ |
| 594.19 | Non | 5.2745 |  | B_u_ |
| 600.32 | Non | 2.393 |  | A_u_ |
| 615.28 | 9.6969 | Non | Bending (A_1_) in P-O_B_-P and Asymmetric (E) deformation of 3(P-O_NB_) (in [AlO_6_] and Na^+^ environment) | B_g_ |
| 618.02 | Non | 19.2358 |  | B_u_ |
| 623.12 | 1.2933 | Non |  | B_g_ |
| 625.38 | Non | 0.1159 |  | A_u_ |
| 640.81 | 13.1038 | Non |  | A_g_ |
| 719.7 | Non | 15.0402 | Symmetric stretching (A_1_) in P-O_B_-P and Symmetric (A_1_) deformation of 3(P-O_NB_) (in [AlO_6_] and Na^+^ environment) | A_u_ |
| 729.4 | Non | 2.4656 |  | B_u_ |
| 736.8 | 135.0469 | Non |  | A_g_ |
| 755 | 0.1149 | Non |  | B_g_ |
| 892.07 | 9.61 | Non | Asymmetric stretching (B_1_) in P-O_B_-P (in [AlO_6_] and Na^+^ environment) | B_g_ |
| 894.74 | Non | 93.7311 |  | B_u_ |
| 911.16 | 2.8296 | Non |  | A_g_ |
| 921.02 | Non | 2.3741 |  | A_u_ |
| 989.78 | Non | 1.4592 | Symmetric stretching (A_1_) of 3(P-O_NB_) and P-O_B_ in Q^1^ (in [AlO_6_] and Na^+^ environment) | B_u_ |
| 994.06 | 0.6725 | Non |  | B_g_ |
| 995.09 | 25.6761 | Non |  | A_g_ |
| 1005.17 | Non | 3.8781 |  | A_u_ |
| 1032.89 | Non | 42.0443 | Symmetric stretching (A_1_) of 3(P-O_NB_) in Q^1^ (in [AlO_6_] and Na^+^ environment) | A_u_ |
| 1033.72 | Non | 1.4029 |  | B_u_ |
| 1054.92 | 261.2154 | Non |  | A_g_ |
| 1066.6 | 4.7098 | Non |  | B_g_ |
| 1073.3 | 81.0847 | Non | Asymmetric stretching (E) of 3(P-O_NB_) in Q^1^ (in [AlO_6_] and Na^+^ environment) | A_g_ |
| 1076.24 | Non | 22.3034 |  | A_u_ |
| 1078.6 | Non | 13.6219 |  | B_u_ |
| 1084.81 | 42.3041 | Non |  | A_g_ |
| 1085.23 | 6.5404 | Non |  | B_g_ |
| 1115.03 | Non | 133.0245 |  | B_u_ |
| 1115.26 | Non | 33.3309 |  | A_u_ |
| 1127.73 | 34.8782 | Non |  | A_g_ |
| 1128.93 | 5.1972 | Non |  | B_g_ |
| 1142.43 | 19.1094 | Non |  | B_g_ |
| 1146.75 | Non | 32.9635 |  | B_u_ |
| 1150.64 | Non | 23.4911 |  | A_u_ |
| 1164.31 | 74.2945 | Non |  | A_g_ |
| 1216.25 | Non | 13.9585 |  | B_u_ |
| 1246.58 | 16.9899 | Non |  | B_g_ |
| 1250.78 | Non | 7.3674 |  | A_u_ |

Non – nonactive, The most intense

**Table S1.6.** Calculated Raman and IR vibrations of **cristobalite type AlPO_4_**. (frequencies of intensities < 0.01 and nonactive in Raman and IR have been omitted).

| Frequency (>200)  [cm^-1^] | Intensity Raman | Intensity IR | Assignment to Q^i^ idealized vibrations and P-O_B_-P | Vibrations in crystal (point group calculated as C_2_ (in real D_2_)) |
| --- | --- | --- | --- | --- |
| 206.59 | 0.4341 | 0.002 | Lattice vibrations and librations | A |
| 207.08 | 0.016 | 0.8664 |  | B |
| 208.43 | 0.0216 | 1.2901 |  | B |
| 215.85 | 0.0005 | 0.1095 |  | B |
| 224.15 | 1.3599 | 0.0001 |  | A |
| 238.88 | 28.7929 | 0.0043 |  | A (B_1_) |
| 309.38 | 0.7479 | 0.3807 | Symmetric bending (E) of Q^0^ (in [AlO_4_] environment) | A |
| 317.57 | 0.3301 | 0.3794 |  | A |
| 337.61 | 0.0038 | 0.0254 |  | B |
| 338.4 | 3.5727 | 0.2273 |  | A |
| 351.35 | 5.9843 | 7.4438 |  | A |
| 355.75 | 19.7411 | 1.3666 |  | A (B_1_) |
| 363.38 | 0.3226 | 0.4522 |  | A |
| 365.42 | 0.5199 | 0.0789 |  | A |
| 376.21 | 0.0009 | 0.0109 | Asymmetric deformation (F_2_) of Q^0^ (in [AlO_4_] environment) | B |
| 388.39 | 0.1994 | 0.9559 |  | B |
| 412.94 | 0.0021 | 0.0265 |  | B |
| 422.33 | 0.0271 | 0.0222 |  | A |
| 427.51 | 2.555 | 30.2095 |  | B (B_3_) |
| 462.42 | 15.2378 | 0.1659 |  | A (A_1_) |
| 469.67 | 4.4684 | 20.6533 |  | B (B_2_) |
| 474.92 | 0.5881 | 20.4627 |  | A (B_1_) |
| 551.52 | 0.0015 | 0.0652 |  | A |
| 552.31 | 0.0074 | 4.0033 |  | A |
| 600.72 | 0.011 | 2.9673 |  | B |
| 712.05 | 2.5951 | 9.9214 |  | B |
| 714.22 | 2.9436 | 0.0041 |  | A |
| 715.34 | 0.4464 | 1.2591 |  | A |
| 724.04 | 0.0018 | 0.018 |  | B |
| 725.93 | 0.398 | 4.5907 |  | B |
| 735.17 | 0.4248 | 3.1608 |  | B |
| 1097.41 | 25.6507 | 0.0008 | Asymmetric stretching (F_2_) of Q^0^ (in [AlO_4_] environment) | A (A_1_) |
| 1101.59 | 1.6935 | 13.1801 |  | B (B_2_) |
| 1104.22 | 0.0999 | 0.2217 |  | A |
| 1104.46 | 0.5848 | 4.1623 |  | B |
| 1104.97 | 9.1075 | 93.1303 |  | B (B_3_) |
| 1105.76 | 4.7251 | 100.0602 |  | A (B_1_) |
| 1105.98 | 15.2926 | 85.0608 |  | B (B_2_) |
| 1109.18 | 73.7524 | 0.2146 | Symmetric stretching (A_1_) of Q^0^ (in [AlO_4_] environment) | A (A_1_) |
| 1112.41 | 0.0062 | 0.0584 | Asymmetric stretching (F_2_) of Q^0^ (in [AlO_4_] environment) | B |
| 1112.5 | 0.0021 | 0.0253 |  | B |
| 1134.14 | 0.1693 | 0.2498 |  | A |
| 1135.99 | 0.8376 | 1.6408 |  | A |
| 1136.3 | 0.2318 | 0.7375 |  | A |
| 1217.36 | 5.6057 | 4.3181 |  | B |

Non – nonactive, The most intense

**Vibrations of Q^0^ in the Table S2.6 and S2.7.**

Q^0^ structural units have T_d_ point group and $\Gamma_{\mathrm{osc}}={1A}_{1}+1E + 2F_{2}$ vibrations without translation and rotation. Fig. S.2.6. shows vibrations in Q^0^.


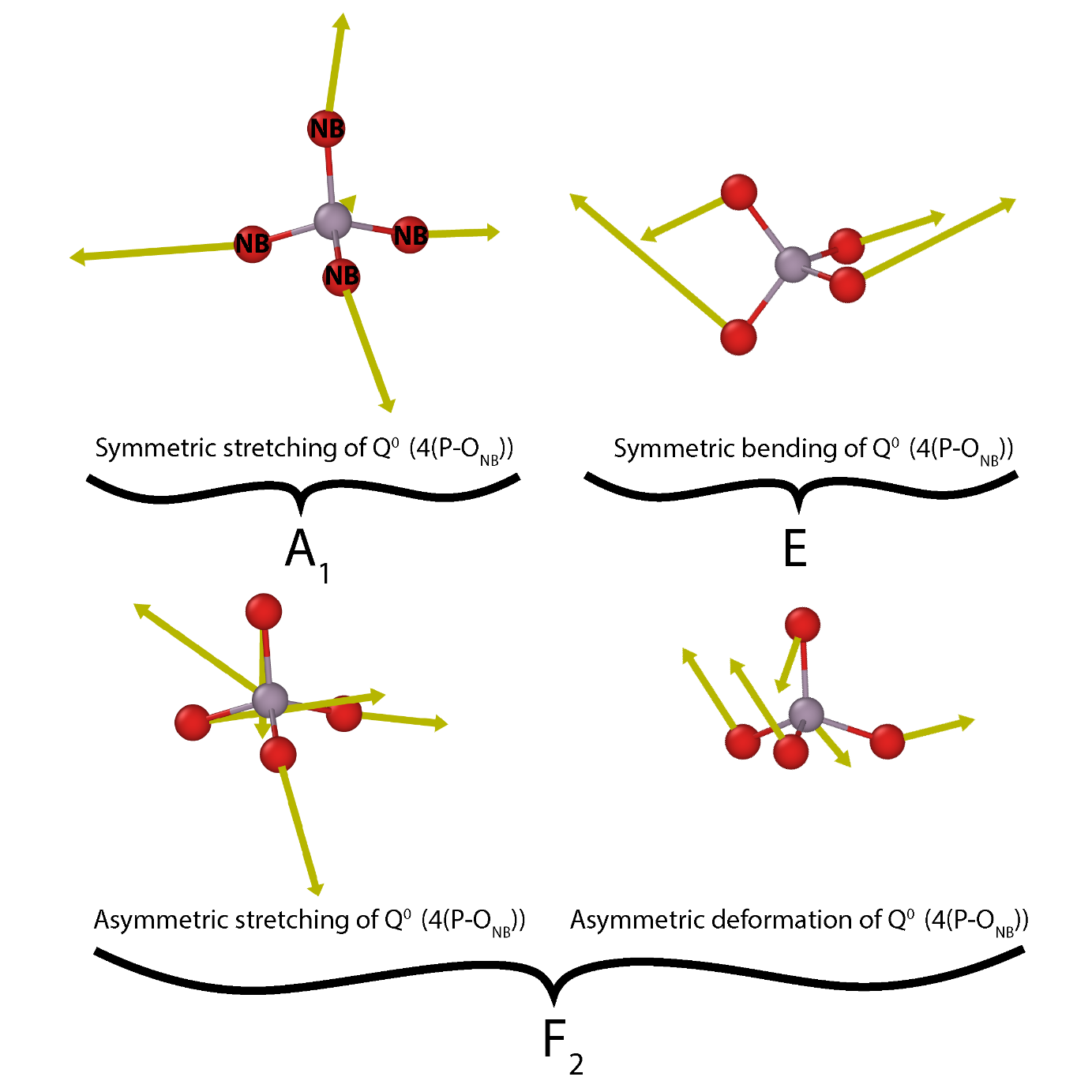


Fig. S1.6. Examples of vibrations in **cristobalite AlPO_4_** assignment to vibrations of idealized Q^0^ unit.

**Table S1.7.** Calculated Raman and IR vibrations of **berlinite AlPO_4_**. (frequencies of intensities < 0.01 and nonactive in Raman and IR have been omitted).

| Frequency (>200)  [cm^-1^] | Intensity Raman | Intensity IR | Assignment to Q^i^ idealized vibrations and P-O_B_-P | Vibrations in crystal (point group calculated as C_1_ (in real D_3_)) |
| --- | --- | --- | --- | --- |
| 246.97 | 0.0001 | 2.8302 | Lattice vibrations and Librations | A |
| 279.89 | 0.1931 | 0.0044 |  | A |
| 280.32 | 0.1847 | 0.0023 |  | A |
| 324.59 | 0.0374 | 0.0001 | Symmetric bending (E) of Q^0^ (in [AlO_4_] environment) | A |
| 359.25 | 1.4289 | 4.1222 |  | A |
| 359.31 | 1.4207 | 4.2352 |  | A |
| 389 | 1.8451 | 1.2443 |  | A |
| 389.73 | 1.8516 | 1.1983 |  | A |
| 401.73 | 7.825 | 0 |  | A |
| 427.9 | 0.001 | 9.4513 |  | A |
| 429.07 | 0.2865 | 3.1402 |  | A |
| 429.45 | 0.2857 | 3.185 |  | A |
| 432.14 | 40.0965 | 0.0003 |  | A (A_1_) |
| 451.4 | 2.0207 | 12.4222 | Asymmetric deformation (F_2_) of Q^0^ (in [AlO_4_] environment) | A (E) |
| 451.85 | 2.0121 | 12.4081 |  | A (E) |
| 468.62 | 0.0001 | 12.9898 |  | A (A_2_) |
| 540.25 | 0.8391 | 0.0017 |  | A |
| 540.47 | 0.8371 | 0.0022 |  | A |
| 636.4 | 0.4427 | 0.4417 |  | A |
| 636.49 | 0.4335 | 0.4336 |  | A |
| 687.74 | 0.0003 | 5.3202 |  | A |
| 690.31 | 0.3129 | 6.2317 |  | A |
| 690.35 | 0.3156 | 6.2185 |  | A |
| 706.55 | 0 | 0.0135 |  | A |
| 719.91 | 0.2331 | 0.0001 |  | A |
| 740.49 | 0.5523 | 0.4546 |  | A |
| 740.5 | 0.5507 | 0.4593 |  | A |
| 1086.75 | 0.0073 | 0.9892 | Asymmetric stretching (F_2_) of Q^0^ (in [AlO_4_] environment) | A |
| 1089.2 | 5.3895 | 55.7831 |  | A (E) |
| 1089.38 | 5.7309 | 51.464 |  | A (E) |
| 1092.29 | 0.0019 | 78.7807 |  | A (E) |
| 1093.16 | 3.4686 | 0.818 |  | A |
| 1095.16 | 2.2729 | 18.7468 |  | A (E) |
| 1095.37 | 2.5068 | 16.5056 |  | A (E) |
| 1102.66 | 70.9074 | 0.0111 | Symmetric stretching (A_1_) of Q^0^ (in [AlO_4_] environment) | A (A_1_) |
| 1121.69 | 1.1556 | 4.0357 |  | A |
| 1121.79 | 1.1316 | 3.9268 |  | A |
| 1217.39 | 8.0801 | 0.0328 | Asymmetric stretching (F_2_) of Q^0^ (in [AlO_4_] environment) | A |
| 1217.54 | 8.0733 | 0.0334 |  | A |

Non – nonactive, The most intense

**Table S2.1.** Phase composition of the sample mainly containing A-Al(PO_3_)_3_ obtained from Rietveld refinement.

| No. | Compound | Content [wt%] | COD database ID |
| --- | --- | --- | --- |
| 1 | A-Al(PO_3_)_3_ | 99.3 | 1010266 |
| 2 | α-Cristobalite type AlPO_4_ | 0.7 | 1532548 |

**Table S2.2.** Phase composition of the sample mainly containing aluminum cyclohexaphosphate obtained from Rietveld refinement.

| No. | Compound | Content [wt%] | COD database ID |
| --- | --- | --- | --- |
| 1 | Aluminum cyclohexaphosphate  -Al(PO_3_)_3_ | 84.6 | 2225399 |
| 2 | A-Al(PO_3_)_3_ | 15.4 | 1010266 |

**Table S2.3.** Phase composition of the sample mainly containing NaAlP_2_O_7_ obtained from Rietveld refinement.

| No. | Compound | Content [wt%] | COD database ID |
| --- | --- | --- | --- |
| 1 | NaAlP_2_O_7_ | 81.5 | 8103838 |
| 2 | α-Berlinite (AlPO_4_) | 9.9 | 9006404 |
| 3 | α-Cristobalite type AlPO_4_ | 5 | 1532548 |
| 4 | α-Al_2_O_3_ | 3.6 | 9008081 |

**Table S2.4.** Phase composition of the sample mainly containing cristobalite type AlPO_4_ obtained from Rietveld refinement.

| No. | Compound | Content [wt%] | COD database ID |
| --- | --- | --- | --- |
| 1 | α-Cristobalite type AlPO_4_ | 67.2 | 1532548 |
| 2 | α-Al_2_O_3_ | 15.6 | 9008081 |
| 3 | α-Berlinite (AlPO_4_) | 9.6 | 9006404 |
| 4 | A-Al(PO_3_)_3_ | 7.6 | 1010266 |

**Table S2.5.** Phase composition of the sample mainly containing berlinite obtained from Rietveld refinement.

| No. | Compound | Content [wt%] | COD database ID |
| --- | --- | --- | --- |
| 1 | α-Berlinite (AlPO_4_) | 56.8 | 9006404 |
| 2 | α-Cristobalite type AlPO_4_ | 21.5 | 1532548 |
| 3 | α-Al_2_O_3_ | 11.4 | 9008081 |
| 4 | A-Al(PO_3_)_3_ | 10.3 | 1010266 |





Fig. S2.1. average- average Raman spectra of eight random points on surface of Al(PO_3_)_3_ sample synthesized in 700 ^o^C and the best matching point to B-Al(PO_3_)_3_ spectra.





Fig. S2.2. IR spectra of Al(PO_3_)_3_ sample synthesized in 700 ^o^C close similar to A-(PO_3_)_3_.
